# Misregulation of *Drosophila* Sidestep Leads to Uncontrolled Wiring of the Adult Neuromuscular System and Severe Locomotion Defects

**DOI:** 10.3389/fncir.2021.658791

**Published:** 2021-06-03

**Authors:** Jaqueline C. Kinold, Marcel Brenner, Hermann Aberle

**Affiliations:** Department of Biology, Institute for Functional Cell Morphology, Heinrich Heine University Düsseldorf, Düsseldorf, Germany

**Keywords:** Drosophila, sidestep, motor axon guidance, muscle innervation, locomotor behaviour

## Abstract

Holometabolic organisms undergo extensive remodelling of their neuromuscular system during metamorphosis. Relatively, little is known whether or not the embryonic guidance of molecules and axonal growth mechanisms are re-activated for the innervation of a very different set of adult muscles. Here, we show that the axonal attractant Sidestep (Side) is re-expressed during *Drosophila* metamorphosis and is indispensable for neuromuscular wiring. Mutations in *side* cause severe innervation defects in all legs. Neuromuscular junctions (NMJs) show a reduced density or are completely absent at multi-fibre muscles. Misinnervation strongly impedes, but does not completely abolish motor behaviours, including walking, flying, or grooming. Overexpression of Side in developing muscles induces similar innervation defects; for example, at indirect flight muscles, it causes flightlessness. Since muscle-specific overexpression of Side is unlikely to affect the central circuits, the resulting phenotypes seem to correlate with faulty muscle wiring. We further show that mutations in *beaten path Ia (beat)*, a receptor for Side, results in similar weaker adult innervation and locomotion phenotypes, indicating that embryonic guidance pathways seem to be reactivated during metamorphosis.

## Introduction

Excitatory motor neurons in the central nervous system and their peripheral target muscles are connected in a precise wiring pattern. This neuromuscular system is established during embryonic development and it is essential for all the coordinated motor behaviours, later in life. The search for molecular cues that regulate the formation of these precise connexions led to the discoveries of secreted and membrane-attached protein families that either attract or repel motor axons (Tessier-Lavigne and Goodman, 1996). While the molecular functions and mutant phenotypes of these guidance molecules have been well-characterised during embryonic development in the last decades (Tessier-Lavigne and Goodman, 1996; Bashaw and Klein, 2010), less is known about their functions during metamorphosis of holometabolous organisms. In *Drosophila melanogaster*, most larval muscles are histolysed during pupation. Adult muscles develop from cellular progenitors that have been set aside in an undifferentiated state during embryogenesis (Bate et al., 1991; Roy and VijayRaghavan, 1999). However, different muscles develop from different pools of stem cells (Gunage et al., 2017). First, abdominal muscles develop from clusters of persistent Twist-expressing cells associated with larval segmental nerves (Currie and Bate, 1991). Second, thoracic muscles and leg muscles develop from Twist-expressing adepithelial cells in imaginal discs (Bate et al., 1991; Broadie and Bate, 1991). Third, the dorsal longitudinal flight muscles develop from a few persisting larval muscles that fuse with the surrounding muscle precursors (myoblasts) (Fernandes et al., 1991).

Prior to the onset of adult muscle development, larval neuromuscular junctions (NMJs) are dismantled but most motor neurons survive and re-grow the axons towards adult targets (flight muscles or abdominal muscles) (Tissot and Stocker, 2000). However, in legless *Drosophila* larvae, only a small number of leg motor neurons are actually born in the embryo and the majority develops *de novo* from larval neuroblasts (NBs) (Baek and Mann, 2009; Brierley et al., 2012). Although the development and projection of leg motor neurons have been described (Consoulas et al., 2002; Baek and Mann, 2009; Brierley et al., 2012; Enriquez et al., 2015) and the pattern of leg muscles is known (Miller, 1950; Soler et al., 2004; Maqbool et al., 2006), it is still unclear, which central circuits coordinate specific leg movements. Largely unknown are also the guidance factors that establish the adult neuromuscular pattern. Amongst classical axon guidance molecules, only Semaphorins and their receptors, plexins have been functionally examined in the imago (Syed et al., 2016). Downregulation of Semaphorin-1a or Plexin-A in leg motor neurons using transgenic RNA interference (RNAi) led to defasciculation defects in the leg nerve and axonal stalls at the level of femur muscles. These flies showed irregular footprint patterns and leg dragging phenotypes on carbon soot-coated glass slides, suggesting that these genes are required for the establishment of functional motor circuits (Syed et al., 2016).

Sidestep (Side) is an axon guidance molecule that functions as a potent attractant for motor axons during embryogenesis (Sink et al., 2001; de Jong et al., 2005). As a member of the Ig-superfamily, it contains five extracellular immunoglobulin domains and a transmembrane domain followed by a relatively short cytoplasmic domain with no identified sequence motifs or binding partners (Sink et al., 2001). The expression pattern of Side is quite unique and highly dynamic, positioning it in substrates at or ahead of growth cones (Siebert et al., 2009). Side is recognised by Beaten path Ia (Beat) that is expressed in all motor neurons (Fambrough and Goodman, 1996; Siebert et al., 2009). In the absence of Side, motor axons show reduced growth rates, fail to defasciculate into their target regions, and migrate in aberrant directions, resulting in muscle fibres carrying NMJs at aberrant positions or lacking them entirely (Kinold et al., 2018). This miswiring defect causes major locomotion impairments in larvae, including reduced crawling speeds and “arching” off the substrate (Kinold et al., 2018). Despite the fact, that ~30% of muscle fibres completely lack NMJs, mutant larvae still manage to climb the steep walls and initiate pupation. Since most of the body wall muscles are histolysed during metamorphosis, it is not known if Side has any function in the establishment of the adult neuromuscular systems or if it is functionally replaced by one of its seven paralogs (Li et al., 2017).

We now find that *side* mutant escapers appear physically weak and have a reduced life span. They easily drown in the food or stick to wet surfaces. Adult motor nerves project along aberrant pathways, and multi-fibre muscles receive abnormal or in some cases, there is even no innervation. This leads to an inability to coordinate leg movements and unstable gait. Miswiring also affects other leg-based behaviours. Similarly, overexpression of Side in all muscles during the development results in erroneous innervation of indirect flight muscles. These results show that misregulation of motor axon guidance molecules cause severe problems with coordinated movements in otherwise viable animals.

## Materials and Methods

### Genetics and Fly Stocks

Flies were kept on standard corn meal food in yeasted vials at 25°C. The *side*^*C*137^and *side*^*I*1563^ alleles were isolated in an EMS mutagenesis screen for recessive mutations on the third chromosome (Aberle et al., 2002). Isogenic *w*^1118^*;;ShGFP* (insertion 7A on III. chr.), also called CD8-GFP-Sh (Zito et al., 1999), was used as a control strain. Side GFP exon trap line and all other GFP exon trap lines listed in Table 1 were obtained from a collection of MiMIC protein trap lines (Nagarkar-Jaiswal et al., 2015). In *SideGFP*^*Mi*00149^ (BL#60507), the Mi00149-GFSTF.1 element is inserted between exons 16 and 17 of the *side* locus and leads to a fusion of amino acid 918 in the cytoplasmic domain of Side to a protein linker followed by GFP (913-QPSLN-GGGGS.). The insertion does not cause axon guidance defects or muscle innervation phenotypes in embryos or larvae, respectively. The following driver-lines were used: *Mef2-Gal4* (Ranganayakulu et al., 1996), *Cha-Gal4* (Salvaterra and Kitamoto, 2001) and *OK371-Gal4* (Mahr and Aberle, 2006). Effector lines include *UAS-Side*, insertion 29A on III. chromosome (Sink et al., 2001), *UAS-mCD8GFP* (Lee and Luo, 1999) and *UAS-CD4tdTomato* (Han et al., 2011). Other lines can be ordered from Bloomington or VDRC and are listed in Table 1.

**Table 1:** Used fly lines ordered from the Bloomington or VDRC stock centres.

**Fly line**	**Number**
*beat^3^*	BL# 4748
*beat^*C*163^*	BL# 4742
*UAS-myrRFP*	BL# 7118
*Zfh1-Gal4*	BL# 25351
*VGlut[MI04979-lexA:QFAD]*	BL# 60314
*LexAop2-mCD8::GFP*	BL# 32203
*UAS-Side-RNAi*	VDRC# 1283
*UAS-Beat-RNAi*	VDRC# 46815
*UAS-Dicer (II. Chr.)*	BL# 24650
*UAS-Dicer (III. Chr)*	BL# 24651

### Preparation and Immunohistochemistry of Imaginal Discs

Wandering third instar larvae were collected and transferred into phosphate-buffered saline (PBS). Larvae were dissected in PBS and fixed in 3.7% formaldehyde for 15 min. Larval fillets were incubated overnight with primary antibodies at 4°C on a rocking platform. Imaginal discs were prepared using forceps (Style 5, Inox 08, Dumont, Switzerland) on a dissection plate prepared with Sylgard (Roland Vetter LaborbedarfOHG, Ammerbuch). For live imaging, discs were directly mounted on 1 × PBS and covered by 18 × 18 mm cover slides.

For antibody staining, discs were fixed in 4% paraformaldehyde in PBS for 30 min. After washing three times with PTw (PBS, 0.1% Tween 20), the discs were blocked for 30 min in PTw containing 5% normal goat serum (NGS). Primary antibodies were added and incubated overnight at 4°C. After washing three times with PTw, secondary antibodies were added and incubated for 1 h at room temperature. Discs were washed three times with PTw and cleared in 80% glycerol in PBS. The following primary antibodies were used: rabbit anti-GFP (1:1000, TP401, Acris Antibodies), mouse anti-GFP (1:400, Clones 7.1 and 13.1, Roche, Basel, Switzerland), rabbit anti-Twist (1:400, kindly provided by M. Leptin) (Roth et al., 1989), rabbit anti-Zfh1 (1:3000, kind gift of M. Frasch) (Broihier et al., 1998), mouse anti-Elav [1:40, clone 9F8A9, Developmental Studies Hybridoma Bank (DSHB), Iowa, United States], anti-HRP (1:1000, ICN Biomedicals, Ohio, United States), anti-Repo (1:40, clone 8D12, DSHB, Iowa, United States), anti-Discs large [1:100, clone 4F3 (DSHB, Iowa, United States)], anti-Sidestep (1:20, clone 9B8, DSHB, Iowa, United States), and Texas Red-coupled phalloidin (1:1000, T7471, Invitrogen, United States). Secondary antibodies include goat anti-mouse Alexa Fluor 488, goat anti-rabbit Alexa Fluor 488, goat anti-rabbit Cy3, and goat anti-mouse Cy3 (all 1:500, Jackson Immuno Research, West Grove, PA, United States).

### Preparation of Developing Legs for Live Imaging

To image innervations of the adult leg musculature legs were prepared from pupae as described earlier (Weitkunat and Schnorrer, 2014). Briefly, pupae (~100 hAPF) were immobilised on double-sided adhesive tape and the pupal case was removed using forceps (Style 5, Inox08, Dumont, Switzerland). Pharate adults were washed in cold PBS and legs were removed using scissors (No. 15018-10, Fine Science Tools, CA, United States). Legs were transferred into 70% glycerol in PBS on microscope slides, covered by 18 × 18 mm cover slides and imaged immediately.

For imaging *SideGFP*^*Mi*00149^ expression and muscle innervation in *ShGFP* and side mutants in developing legs, pupae at different stages were collected and washed in PBS. Developing legs were dissected on custom-made Sylgard plates using forceps (Style 5, Inox 08, Dumont, Switzerland). After fixation in 4% paraformaldehyde in PBS for 30 min, the legs were washed three times with PBS. The cuticle was then removed in a drop of PBS on Sylgard plates using forceps. For live imaging, the developing legs were mounted on PBS covered by 18 × 18 mm cover slides.

### Immunohistochemistry of Adult Flight Musculature

Adult flight muscles were prepared and stained as described (Schnorrer et al., 2010). Briefly, 3 to 6-days-old adult flies were collected and the head, legs, and abdomen were removed. Thoraces were fixed two times for 10 min in 4% paraformaldehyde in relaxing solution [20 mm phosphate buffer (pH 7), 5 mm MgCl, 5 mm EGTA]. Fixed thoraces were split by sagittal dissection. Tissues were blocked for 20 min in PBS containing 3% normal goat serum (NGS, Jackson ImmunoResearch, West Grove, PA, United States). After washing, the rabbit anti-Ankyrin2-XL antibodies (Koch et al., 2008) were diluted in 1:1000 PTx (PBS, 0.2% Triton-X100) and added for 1 h at room temperature or overnight at 4°C. Thoraces were rinsed and incubated with goat anti-rabbit secondary antibodies conjugated with Cy3 (1:500, Jackson ImmunoResearch, West Grove, PA, United States) for 1 h. After washing, thoraces were cleared in PBS/70% glycerol and covered by 22 × 22 mm cover slides for imaging.

### Microscopy and Image Quantification

**Figure 1:**
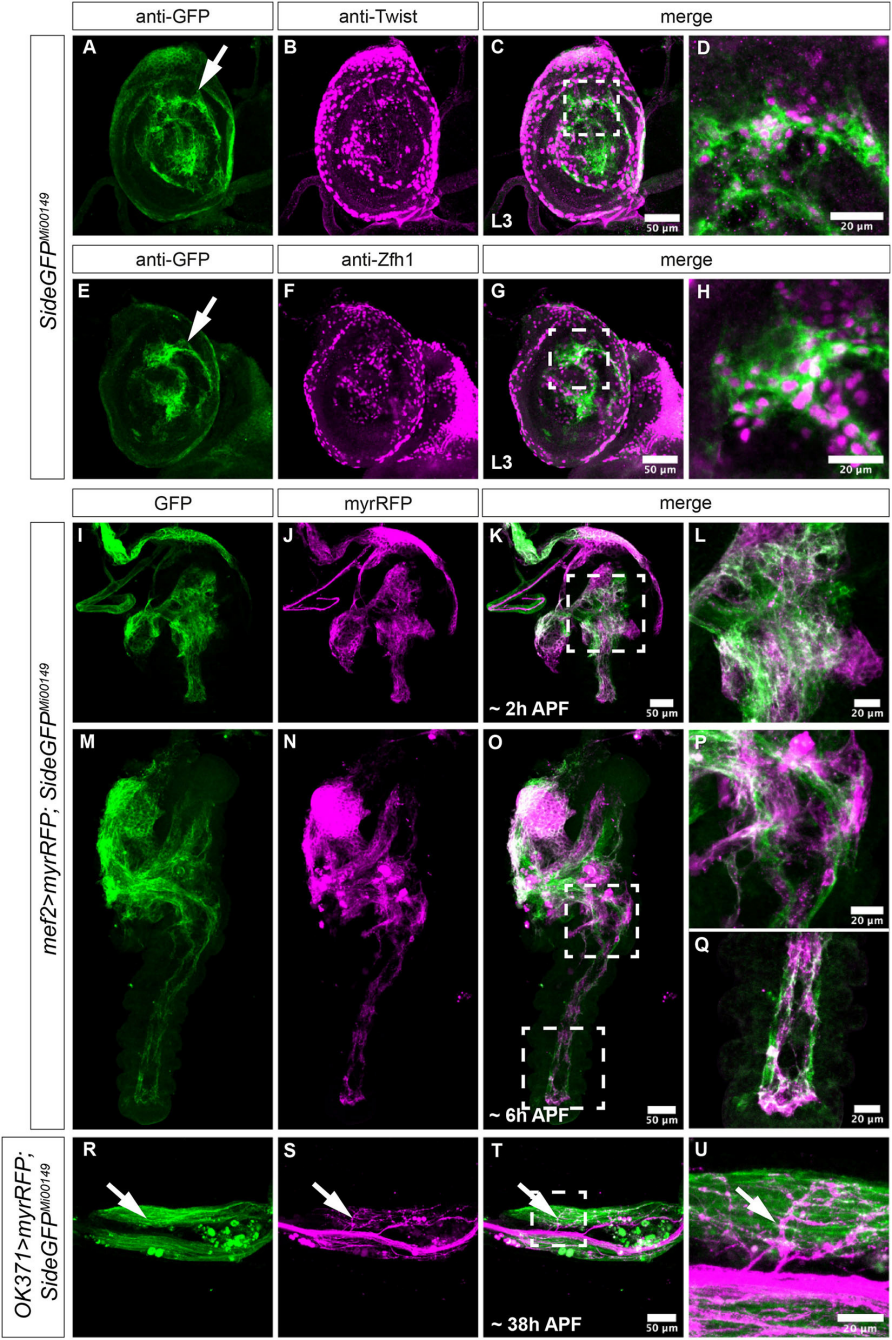
Expression of Side in *Drosophila* imaginal discs. **(A–U)** Projections of confocal micrographs acquired from dissected leg imaginal discs of late *SideGFP*^*Mi*00149^ third instar larvae and early pupae of the indicated genotypes. White frames mark regions enlarged in new micrographs in **D,H,L,P,Q,U**. **(A,E)** In legdiscs, GFP is detected mainly in central patches of different brightness that are concentrically arranged (arrows). **(A–D)** Side-GFP partially but not completely overlaps with Twist-expressing myoblasts in leg discs. **(E–H)** Central regions of leg discs are enriched for Zfh1-positive cells, which co-localise with Side-GFP. **(I–Q)** At 2 and 6 h after puparium formation (APF), muscle precursors expressing myrRFP under control of *Mef2-Gal4* (magenta) largely overlap with Side-GFP (**M–O** are tile scans). **(R–U)** Motor axons expressing myrRFP (magenta) driven by *OK371-Gal4* innervate Side-GFP-expressing muscles (green, arrows) in the femur of a pharate SideGFP^Mi00149^ pupa. Genotypes: *yw;*+*; SideGFP*^*Mi*00149^, *w;Mef2-Gal4/UAS-myr-RFP,SideGFP*^*Mi*00149^*, w;OK371-Gal4/UAS-myr-RFP; SideGFP*^*Mi*00149^. Scale bars: **C,G,K,O,T** 50 μm; **D,H,L,P,Q,U** 20 μm.

**Figure 2:**
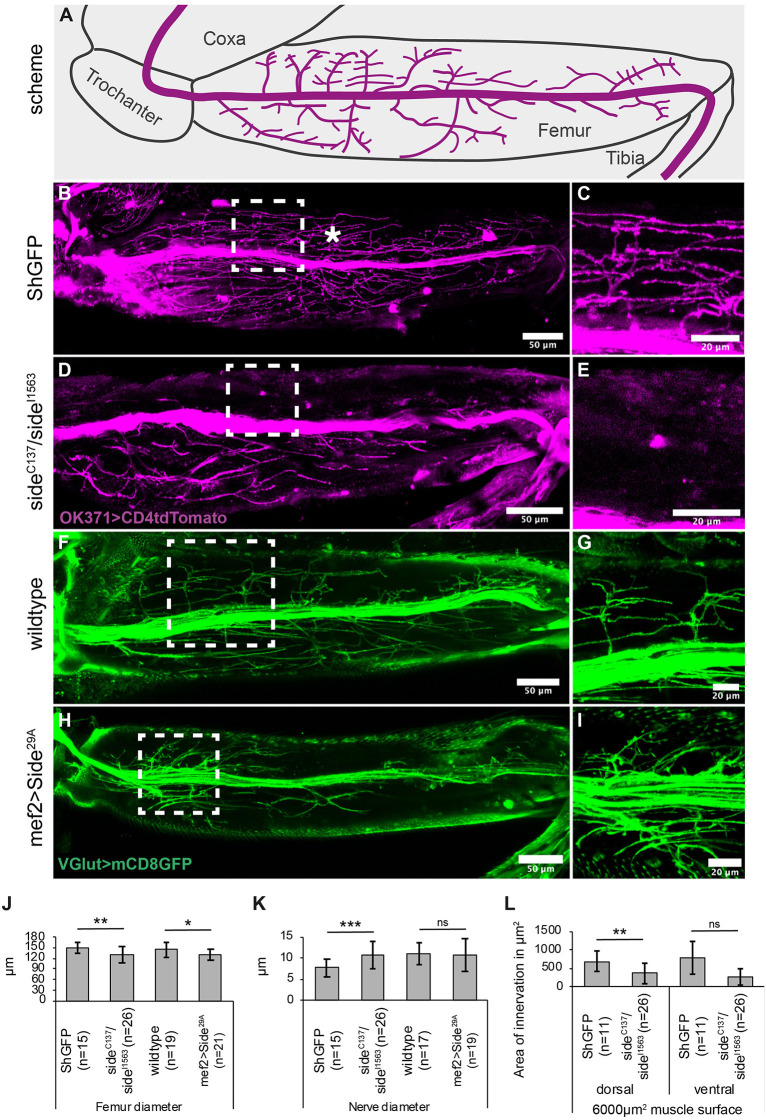
Projection errors of leg nerves in *side* mutant and overexpressing Side flies. **(A)** Scheme of a tentative projection pattern of motor nerves (magenta) in the femur. **(B,C)** Confocal projections of the femur acquired through the translucent cuticle of a *ShGFP* control animal, expressing CD4tdTomato (magenta) under control of OK371-Gal4 in motor axons. The leg nerve forms a central bundle and sprouts numerous primary and secondary branches that evenly cover the dorsal tilm (asterisk) and ventral tidm muscles. **(D,E)** Homozygous *side* mutant pharate adults display a thicker central bundle and less branches reach the tilm muscle. **(F,G)** Motor axons expressing mCD8GFP (green) under the control of *VGlut-LexA* in a wild-type background. **(H,I)** Motor axons expressing mCD8GFP (green) under control of *VGlut-LexA* in pharate adults overexpressing Side in developing muscles using *Mef2-Gal4*. Motor axons show excessive branching in proximal regions of the tilm and tidm muscles. **(J–L)** Quantitative analysis of femur and leg nerve diameters **(J,K)** and area of innervation of tilm and tidm muscles **(L)** in control, *side* mutant and overexpressing Side flies. Femurs are slightly thinner **(J)** and leg nerves are thicker **(K)** on an average in *side* mutants. Dorsal tilm muscles evaluated by Student's *t*test but not ventral tidm muscles evaluated by Mann–Whitney-*U*-test receive less innervation in *side* mutants compared to controls (*p* < 0.119) **(L)**. Data are means ± SD, *n* = number of femurs. Statistical significance is calculated using two-tailed Student's *t*-test or Mann–Whitney-*U*-test: ***P < 0.001; **P < 0.01, *P < 0.05; ns, not significant. White frames mark regions enlarged by new micrographs in **C,E,G,I**. Genotypes: *w;*+*;OK371-Gal4, ShGFP/UAS-CD4tdTomato, w;OK371-Gal4/*+*;side*^*C*137^*, ShGFP/side*^*I*1563^*, ShGFP, UAS-CD4tdTomato, w;VGlut*^*Mi*04979^*-LexA/*+*;LexAop-mCD8GFP/*+*, w;VGlut*^*Mi*04979^*-LexA/Mef2-Gal4;LexAop-mCD8GFP/UAS-Side*^29*A*^. Scale bars: **B,D,F,H** 50 μm; **C,E,G,I** 20 μm.

Microscopic images were acquired on a laser-scanning confocal microscope (LSM710, Carl Zeiss MicroImaging, Jena, Germany) using air objectives (20x/0.8 and 40x/0.95 Korr Plan Apochromat). Images (1,024 × 1,024 pixel, line averaging 2) were processed using Fiji is just ImageJ software (Schindelin et al., 2012). Figures show maximum intensity projections of several z sections. The following figures are tile scans: Figures 1L–N, 2B,D,F,H, **4A,B,H,K,O**, **6A,C**; Supplementary Figure 1A.

Terminal axon branches were counted using the multi-point tool in Fiji is just ImageJ (Schindelin et al., 2012). To quantify the average number of terminal branches, a representative muscle area of 100 × 100 μm per hemi-thorax was selected. In flies overexpressing Side in muscles, branch numbers were assessed in two separate 100 × 100 μm areas containing either high or low innervation. In order to determine the number of axonal branches in ventral vs. dorsal muscle regions in both wild-type and Side overexpressing flies, an area of 25 × 100 μm was selected at the ventral nerve entry site and the opposing dorsal side of a single muscle fibre.

Areas of innervation of femur musculature were analysed using threshold application in Fiji is just ImageJ (Schindelin et al., 2012). To quantify the average area of innervation, two representative areas (60 × 100 μm) in the middle of the femur of were selected, one in the dorsal part and one in the ventral part of the femur. For indirect flight muscles, representative areas (60 × 100 μm) were selected by the experimenter by eye.

### Videography

High-speed movies of walking and flying adult flies were collected using a 1.3 megapixel high-speed imaging camera (PhotronFastcam Mini UX100 Model 800K/M1) (VKT, Pfullingen, Germany) equipped with a macro zoom lens (computar macro zoom 0.3x, 1:4.5 coupled with a Cosmicar/Pentax X2 Extender). For recording walking behaviour, flies were allowed to walk over cover slides and were recorded with 125 frames per second (fps) from a ventral view point. Flies were allowed to voluntarily take off and were recorded from lateral views. The frame rate was 5,000 fps. Flies were fixed to a minutien pin on the scutum using UV-curing adhesives (UV glue for glass and crystal, Ber-Fix, Berlin, Germany). Movies were filmed from frontal or lateral views having 5,000 to 10,000 fps. The number of wing beats per second and the amplitude of wings were analysed using Tracker (version 4.11.0, Douglas Brown, Open Source Physics). Images were saved on a Lenovo 2447/W530 notebook and processed with PhotronFastcam Viewer software (version 3.52; VKT, Pfullingen, Germany) for data export to an iMac PC (Apple Inc., Cupertino, CA, United States). Time series were assembled and edited using Fiji is just ImageJ (Schindelin et al., 2012).

### Locomotion Assays

For all locomotion assays, male flies were collected on the day of hatching. The assays were carried out on the next day to rule out any intoxication by carbon dioxide, which might lead to abnormal motion behaviours.

#### Leg Print Assay

Wings of males per genotype were removed the day before the assay, as previously described (Maqbool et al., 2006). Briefly, microscope slides were coated with carbon soot, and each fly was allowed to walk over two slides. Tracks were imaged using a light microscope (Axio Imager M2; Carl Zeiss MicroImaging, Jena, Germany) equipped with a CCD camera (AxiocamMRm, Carl Zeiss MicroImaging, Jena, Germany). The ability to walk over the microscope slides was analysed based on the position of tarsus prints. Step length (distance between two tarsi prints) of the mesothoracic (second) leg was measured for 10 steps per male fly using AxioVision software (Carl Zeiss MicroImaging, Jena, Germany). Average length of tarsal prints of the metathorcaic (third) leg was similarly determined by averaging ten prints per male. Mean values of each fly were summed up to calculate the mean for each per genotype.

#### Grooming Assay

To test if flies are able to clean their body, single flies were covered with Reactive Yellow 86 dust (Organic Dyestuffs Corp., Concord, NC, United States) as previously described (Seeds et al., 2014). Flies were assayed in small plastic cages with a nylon mesh at the bottom. Pictures were acquired at 0, 4, 30, and 60 min using a stereomicroscope M80 (Leica Microsystems, Wetzlar, Germany) equipped with a CCD camera IC80HD (Leica Microsystems, Wetzlar, Germany) and Debut Video Capture software.

#### Climbing Assay

To test if flies are able to climb, the negative geotaxis assay was carried out as primarily described (Chaudhuri et al., 2007). Flies were transferred into two empty tubes and were stuck together with a sticky tape. Flies were tapped to the bottom of one tube and the number of flies that were able to climb 7 cm in 7 and 15 s was counted. Ten replications were performed for each cohort. For analysis, the mean of flies passing the mark per cohort and thereof the mean per genotype was calculated.

#### Dropping Assay

The ability to fly was tested using a dropping assay described by Newquist et al. (2013). Three males were dropped at the same time from one vial onto the lab bench. Each fly that escaped by flight was counted as flyer.

#### Island Assay

To test if flies are able to take off from a flat substrate, flies were placed on an island surrounded by water containing a detergent (Schmidt et al., 2012). Take-off behaviour was monitored for 2 min using a Discovery Deluxe VMS-004 video camera (Veho Europe, Southampton, UK) and a test version of the Debut Video Capture software (NCH Software, Greenwood Village, CO, United States). Vanished flies were counted manually on the computer screen after 10 s and 120 s.

### Statistical Analysis

Statistical diagrams display the mean and SD of the measurements using Microsoft Excel. Data sets were tested for normal distribution using the Kolmogorov–Smirnov test with Lilliefors correction. *P*-values of two group comparisons were determined using a two-tailed, unpaired Student's *t*-test for normal distributed data sets or were calculated using a two-tailed Mann–Whitney *U*-test for abnormal distributed data sets. Comparison of *p*-values of more than two groups was determined using a one-way ANOVA associated with a Dunnet's *post-hoc* test (summarized in Table 2). *P*-values are shown in graphs as follows: ^***^p ≤ 0.001, ^**^p ≤ 0.01, ^*^p ≤ 0.05, ns = not significant. Diagrams depicting percentages show total numbers. *P*-values were determined using a two-tailed Fisher exact test (^***^p ≤ 0.001, ^**^p ≤ 0.01, ^*^p ≤ 0.05, ns = not significant).

**Table 2:** Statistical summary of one-way ANOVA with associated Dunnet's *post-hoc* test.

**Figure**	**Description**	**Output**	***P*-values**
3L	Step length	*F*_(4,94)_ = 51.22	ANOVA ≤ 0.0001, ShGFP vs. Side^I1563^/Side^C137^ = 0.903, ShGFP vs. Mef2 > Side^29A^ ≤ 0.0001, ShGFP vs. Mef2 > SideRNAi ≤ 0.0001, ShGFP vs. Mef2 > SideRNAi + Dicer = 0.0095
	Length of metathoracic leg print	*F*_(4,94)_ = 6.145	ANOVA = 0.0002, ShGFP vs. Side^I1563^/Side^C137^ = 0.0001, ShGFP vs. Mef2>Side^29A^ = 0.0008, ShGFP vs. Mef2>SideRNAi = 0.2120, ShGFP vs. Mef2>SideRNAi+Dicer = 0.0226
3M	7 s	*F*_(4,33)_ = 43.98	ANOVA ≤ 0.0001, ShGFP vs. Side^I1563^/Side^C137^ ≤ 0.0001, ShGFP vs. Mef2>Side^29A^ ≤ 0.0001, ShGFP vs. Mef2>SideRNAi ≤ 0.0001, ShGFP vs. Mef2>SideRNAi+Dicer = 0.0036
	15 s	*F*_(4,33)_ = 37.16	ANOVA ≤ 0.0001, ShGFP vs. Side^I1563^/Side^C137^ ≤ 0.0001, ShGFP vs. Mef2>Side^29A^ ≤ 0.0001, ShGFP vs. Mef2>SideRNAi ≤ 0.0001, ShGFP vs. Mef2>SideRNAi+Dicer = 0.0166
4Q	Terminal branches	*F*_(3,34)_ = 15.54	ANOVA ≤ 0.0001, ShGFP vs. Side^I1563^/Side^C137^ = 0.6851, ShGFP vs. Mef2>Side^29A^ more branches ≤ 0.0001, ShGFP vs. Mef2>Side^29A^ less branches = 0.4824
5E	Maximum	*F*_(2,14)_ = 4.334	ANOVA = 0.0343, ShGFP vs. Side^I1563^/Side^C137^ = 0.0253, ShGFP vs. Mef2>Side^29A^= 0.8934
	Minimum	*F*_(2,14)_ = 0.8091	ANOVA = 0.4650, ShGFP vs. Side^I1563^/Side^C137^ = 0.5421, ShGFP vs. Mef2>Side^29A^= 0.9234
5F	Wing beats	*F*_(2,27)_ = 3.600	ANOVA = 0.0411, ShGFP vs. Side^I1563^/Side^C137^ = 0.1295, ShGFP vs. Mef2>Side^29A^= 0.0275
7A	7 s	*F*_(3,22)_ = 23.14	ANOVA ≤ 0.0001, ShGFP vs. Beat^3^/Beat^C163^ ≤ 0.0001, ShGFP vs. OK371>BeatRNAi = 0.0038, ShGFP vs. OK371>BeatRNAi+Dicer = 0.2033
	15 s	*F*_(3,22)_ = 20.97	ANOVA ≤ 0.0001, ShGFP vs. Beat^3^/Beat^C163^ ≤ 0.0001, ShGFP vs. OK371>BeatRNAi = 0.0003, ShGFP vs. OK371>BeatRNAi+Dicer = 0.9233
7L	Step length	*F*_(3,76)_ = 14.27	ANOVA ≤ 0.0001, ShGFP vs. Beat^3^/Beat^C163^ = 0.0457, ShGFP vs. OK371>BeatRNAi ≤ 0.0001, ShGFP vs. OK371>BeatRNAi+Dicer ≤ 0.0001
	Length of metathoracic leg print	*F*_(3,76)_ = 7.423	ANOVA = 0.0002, ShGFP vs. Beat^3^/Beat^C163^ = 0.0003, ShGFP vs. OK371>BeatRNAi = 0.0003, ShGFP vs. OK371>BeatRNAi+Dicer = 0.0293

## Results

Motor axon guidance phenotypes occurring in *sidestep* (*side*) mutant embryos are irreversible and lead to permanent innervation errors in larvae (Siebert et al., 2009; Kinold et al., 2018). Since the majority of these muscles are histolysed during pupation, it is currently not known to which extent the embryonic guidance systems are re-used to wire the adult musculature. In principle, we would expect that Side is re-expressed during metamorphosis but since it has 7 paralogs (Li et al., 2017); it is also possible that other family members replace Side, functionally in pupae.

For an initial analysis of Side expression, we chose leg imaginal discs, as they are already associated with adult motor nerves in wandering third instar larvae (Brierley et al., 2012; Venkatasubramanian et al., 2019). Motor axons reach the discs by exiting the developing suboesophageal ganglion and by migrating along the existing segmental nerves. Then, they project as a single nerve bundle across the central disc areas.

We visualised Side at this stage using the GFP-exon trap line Mi00149-GFSTF (Nagarkar-Jaiswal et al., 2015), hereafter called *SideGFP*^*Mi*00149^, that fuses with the cytoplasmic domain of Side to GFP at amino acid position 918 and does not disrupt protein function (see section Materials and Methods, Supplementary Figure 3). *SideGFP*^*Mi*00149^ was predominantly expressed in central discs areas (leg disc proper), but overall, it did not seem to reflect any ordered cell arrangements (Figures 1A,E). In particular, we were unable to discern any Side-positive axons growing either towards or away from the discs. To identify these Side-expressing cells, we performed antibody co-stainings. In leg discs, muscle progenitors (myoblasts) express high levels of Twist and occupy defined territories that can be assigned to adult leg muscles (Currie and Bate, 1991; Maqbool et al., 2006; Maqbool and Jagla, 2007). *SideGFP*^*Mi*00149^ partially overlapped with these Twist-expressing cells (Figures 1A–D) but prevailed also in more central areas, where Twist was largely absent, indicating that it might be expressed in additional cell populations.

In imaginal discs, myoblasts are derived from stem cell-like adult muscle precursors (AMPs) that are marked by the transcription factor, Zinc finger homeodomain 1 (Zfh1) (Gunage et al., 2014). Zfh1 acts as a transcriptional repressor and functions upstream of Twist by inhibiting its transcriptional activation (Postigo et al., 1999). Staining *SideGFP*^*Mi*00149^ leg discs with anti-GFP and anti-Zfh1 antibodies showed co-expression of Side and Zfh1 in a substantial portion of cells located in central areas (Figures 1E–H). Taken together, in leg imaginal discs, Side is consistently expressed in different populations of muscle precursors but not in the neurons.

During leg disc eversion, *SideGFP*^*Mi*00149^ largely overlapped with developing muscle fibres visualised by myrRFP under control of *Mef2-Gal4* (Figures 1I–Q). When we visualised motor nerves at ~38 h after puparium formation (APF) using *OK371-Gal4* and *UAS-myrRFP*, most, if not all, axonal sprouts were in direct contact with *SideGFP*^*Mi*00149^-expressing muscles (Figures 1R–U). Thus, Side-expressing muscle fibres provide an attractive substrate for outgrowing motor axons.

### Adult *Side* Mutant Flies Show Severe Misinnervation of Leg Muscles

If Side plays a major role in wiring adult motor nerves, we would expect to find lasting projection errors in leg nerves. The femur contains four anatomically and functionally distinguishable multi-fibre muscles (Soler et al., 2004). The most significant ones, the tibia levator muscle (Tilm) and tibia depressor muscle (Tidm), fill large parts of the dorsal and ventral compartment, respectively (Soler et al., 2004; Maqbool and Jagla, 2007; Baek and Mann, 2009).

Since the cuticle of the femur is translucent in late pupae and freshly hatched flies, leg nerves are amenable for confocal imaging using motor neuron-specific *OK371-Gal4* and the fluorescent membrane marker, CD4tdTomato (Mahr and Aberle, 2006; Han et al., 2011). The leg nerve transverses the femur in a central position, keeping a distance of ~60–80 μm from the cuticle and running parallel to the internal tendon (93%, *n* = 15, Figures 2A,B).

The motor nerve sprouts distinct branches that themselves develop numerous secondary branches in the Tilm and Tidm territories (Figures 2A,B). Higher magnification showed distinct presynaptic varicosities and dense coverage of the muscle surface area (Figure 2C). In contrast, the density of axonal branches was reduced in *side* mutants and large muscle areas were partially devoid of presynaptic endings (Figures 2D,E, quantified in Figure 2L). Tilm or Tidm muscles could be affected simultaneously or individually. The phenotypes were thus somewhat variable, as has been observed for larval muscles (Kinold et al., 2018). The leg nerve generally traversed the femur in a straight line but in approximately half of the specimen, it was dislocated to peripheral regions (54%, *n* = 26) (Supplementary Figures 1A,B).

Pro-, meso- and metathroracic legs, also called first (T1), second (T2), and third (T3) legs, were all affected in a similar manner. We also measured if reduced innervation affected femur size. The diameter of the femur was on an average slightly reduced in *side* mutants (130 ± 22 μm compared to 149 ± 14 μm in controls) (Figure 2J). Failure of motor axons to defasciculate into the muscle areas caused on average an increased diameter of the leg nerve in *side* mutants (10.6 ± 3.1 μm compared to 7.7 ± 2.0 μm in controls) (Figure 2K). In addition, we analysed the reduced innervation in *side* mutants on a defined muscle surface area (6,000 μm^2^) in the middle of the femur. With 370 ± 270 μm^2^, *side* mutants showed reduced innervation areas on the dorsal tilm muscles compared to control flies (690 ± 277 μm^2^) (Figure 2L), suggesting that defasciculation errors in *side* mutants cause insufficient innervation.

Since the muscle-specific overexpression of Side leads to innervation and locomotion defects in larvae (Kinold et al., 2018), we also examined these adult flies. To simultaneously mark motor neurons, we used the LexA/LexAop system (Lai and Lee, 2006). When crossed to *LexAop-mCD8GFP*, the effector line *VGlut[MI04979-lexA:QFAD]* (Diao et al., 2015), hereafter called *VGlut-LexA*, showed a similar expression pattern as seen with *OK371*>*CD4tdTomato*. The major leg nerve passed through the femur at a central position, sprouting dorsal and ventral branches (Figures 2F,G). Muscle-specific overexpression of Side drastically altered the innervation pattern, resulting in muscle areas with hyperinnervations, while other areas were completely devoid of innervation (Figures 2H,I). This difference was particularly evident in the proximal-most and distal-most regions of femur muscles. Interestingly, an average number of axons seemed to be able to defasciculate as the diameter of the main nerve did not differ from controls (11 ±4 μm vs. 11 ± 2.5 μm) (Figure 2K).

### Adult Flies Lacking Side Show Severe Leg Coordination Defects During Walking

**Figure 3:**
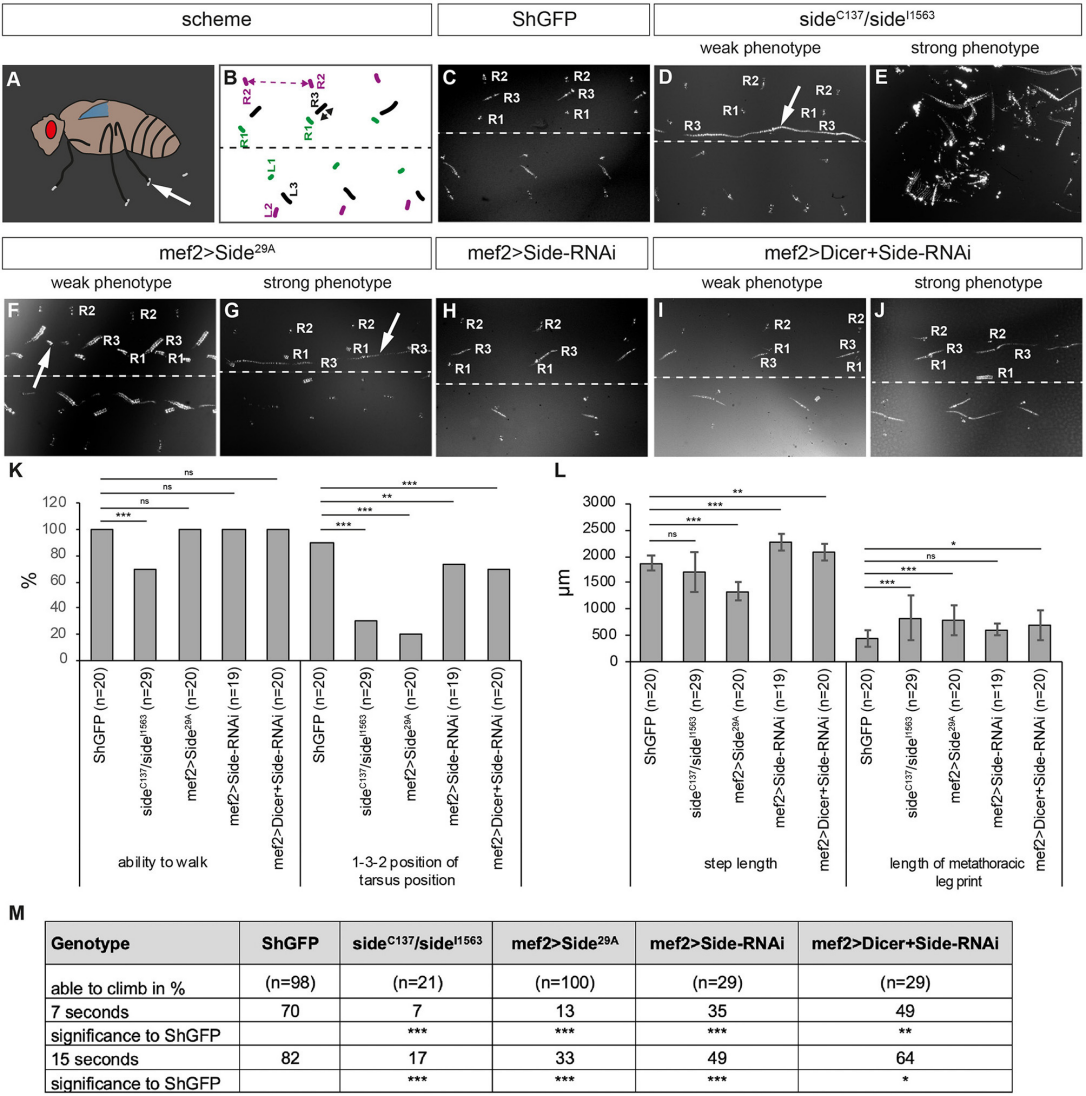
Misinnervation of leg muscles strongly alters walking behaviour. **(A)** Scheme of leg print assay. Arrow marks a leg print. **(B)** Scheme of stereotyped foot print patterns (colour-coded, e.g., R1 = right prothoracic leg, green). Dotted line indicates body midline. Stippled double-headed arrow (magenta), step length of mesothoracic leg. Black double-headed arrow, length of metathoracic tarsus print. **(C–J)** Bright-field images of tarsus prints on carbon soot-coated glass slides. **(C)** Leg print pattern of *ShGFP* control flies. Prints of right legs are labelled from medial to lateral: R1, R3, and R2. **(D,E)** *Side* mutant walking phenotypes vary from dragging R3 at an unusual inner-most position (weak phenotype, arrow in **D**) to being completely unable to walk (strong phenotype, **E**). **(F,G)** Overexpressing Side flies show some minor stumble (weak phenotype, arrow in **F**) or more obvious dragging phenotypes (strong phenotype, arrow in **G**). **(H)** Downregulation of Side using RNAi driven by *Mef2-Gal4* results in no obvious phenotypes. **(I,J)** Enhancing RNAi by Dicer leads to clearly visible prolonged prints of the third leg **(J)**. **(K,L)** Statistical analysis of leg print assays. **(K)** Approximately 30% of *side* mutants (*n* = 29) are not able to walk across the glass slide. Both loss and gain of Side function as well the downregulation of Side result in various percentages, in altered successions in the T1-T3-T2 print pattern. **(L)** Overexpressing Side flies exhibit a shortened step length of the mesothoracic legs (T2-T2), while downregulation of Side using RNAi results in an increased step length compared to controls. **(M)** Results of the climbing assay. Downregulation, loss, or gain of Side leads to a reduced climbing ability of adult flies compared to controls. Data are total number **(K)** or means ± SD **(L+M)**, Fisher exact test **(K)**, one-way ANOVA **(L+M)**: ***P < 0.001, **P < 0.01, *P < 0.5; ns, not significant; n, number of males. Genotypes: *w;*+*;ShGFP, w;*+*;side*^*C*137^*, ShGFP/side*^*I*1563^*, ShGFP, w;*+*;Mef2-Gal4, ShGFP/UAS-Side*^29*A*^, *w;UAS-Side-RNAi/*+*;Mef2-Gal4/*+*;w;UAS-Side-RNAi/*+*;Mef2-Gal4/UAS-Dicer*.

To assess if these innervation phenotypes correlate with locomotion defects, we turned to a leg print assay (Maqbool et al., 2006), which assesses the leg coordination by recording the spatial positions of each leg in a thin layer of carbon soot on a glass slide (Figures 3A,B). During straight forward walks, wild-type flies generally use an alternating tripod gait, resulting in a characteristic print pattern on each side of the body axis (Figures 3B,C). The pro- and mesothoracic legs usually occupy the medial and lateral-most positions, respectively (T1-T3-T2 pattern, Supplementary Movie 1). Compared to *ShGFP* controls, the loss of *side* resulted in a range of phenotypes. Among the weakest phenotypes, we found an inability to raise one leg above the substrate, resulting in long stripes rather than individual prints in the carbon layer (Figure 3D, Supplementary Movie 1). This correlated also with a change in the mediolateral positioning of the legs, with the metathoracic leg now being closest to the midline (T3-T1-T2 pattern). It is important to note that the dragging phenotype was occasionally unilateral and occurred only on one side of the body. Strong phenotypes were represented by stumbling flies, including those that were completely unable to walk over the glass slide (Figure 3E). Quantitative analysis showed that 31% of *side* mutant flies (*n* = 29) failed to walk across the glass slide and 69% (*n* = 20) were unable to keep the T1-T3-T2 pattern (Figure 3K).

Overexpression of Side in muscle precursors using *Mef2-Gal4* produced similar walking phenotypes (Figures 3F,G). However, extreme patterns were not observed and leg dragging was among the strongest phenotypes we noticed in about 30% of the flies (Figure 3G, Supplementary Movie 1). Most flies (70%) showed quite weak phenotypes with misaligned and supernumerary leg prints (Figure 3F). Somewhat unexpectedly, downregulation of Side by RNA inference (RNAi) in developing muscles resulted in almost no discernible print patterns (Figure 3H) but increasing RNAi efficiency by co-expression of Dicer (Dietzl et al., 2007) caused stumbling and leg dragging (Figures 3I,J, for the effectiveness of Side downregulation see Supplementary Figures 3H–J).

Quantitative analysis showed that step length, here the distance between two successive T2 prints, was significantly reduced in overexpressing Side flies (1.3 ± 0.2 mm) but not in *side* mutants (1.7 ± 0.4 mm) when compared to controls (1.9 ± 0.1 mm) (Figure 3L). T3 legs usually leave characteristic dash-like rather than point-like prints (Maqbool et al., 2006; Oyallon et al., 2012). Print length was slightly longer in the mutants compared to isogenic controls (0.8 ± 0.4 mm in *side*, 0.4 ± 0.1 mm in *ShGFP*) (Figure 3L). Most importantly, we observed evident abnormalities in other leg-based behaviours, such as climbing or grooming (Figure 3M, Supplementary Figures 1C–O). These results demonstrate that loss and gain of Side function disturb coordinated movement patterns in adult flies.

To test if Side has a tissue-specific requirement in muscles, we turned to a climbing assay. In this negative geotaxis assay, flies were dropped to the bottom of an empty vial and then allowed to freely climb its walls until they reach a predefined distance (see Materials and methods). Compared to controls, downregulation of *side* in muscles using transgenic RNAi controlled by *Mef2-Gal4* (or *Zfh1-Gal4*) clearly reduced climbing distances both after 7 and 15 s (Figure 3M). These results suggest that reducing Side levels selectively in muscles correlates with reduced climbing abilities. Since we cannot completely rule out a function for *side* in wiring central circuits, our combined loss- and gain-of-function experiments indicate at least that misinnervation of somatic muscles contributes to the observed locomotion defects.

### Overexpression of Side Cause Innervation Defects at Dorsal Longitudinal Flight Muscles

Several adult behaviours, such as feeding or flying are leg-independent in *Drosophila*. In particular, flight is governed by massive indirect flight muscles (IFMs) in the thorax that drive the wing beat indirectly by vibrating the thoracic exoskeleton. Since several IFMs combine to form a functional unit, it is unclear how mutations in *side* might affect flight.

**Figure 4:**
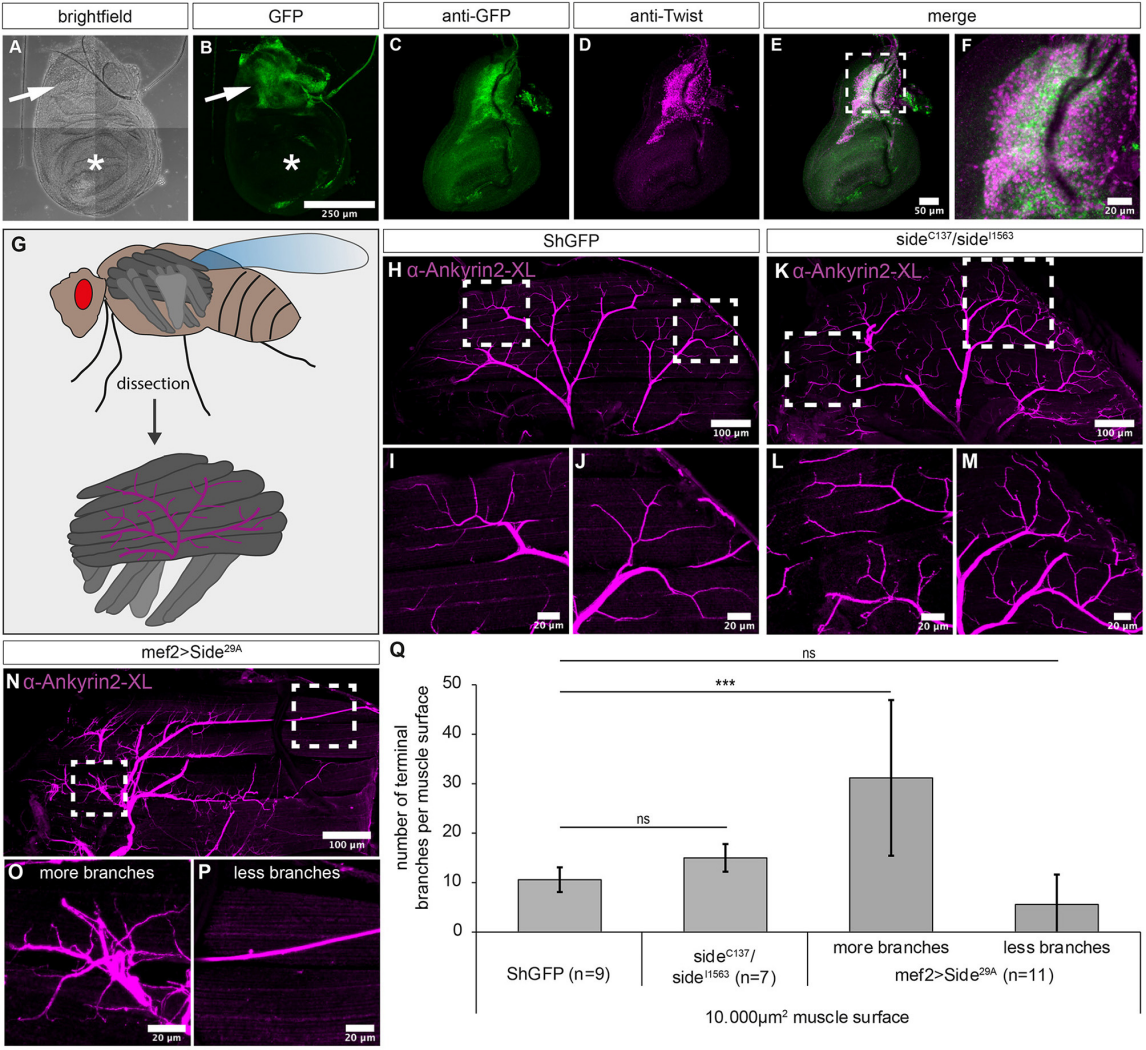
Overexpression of Side causes motor axon targeting errors in indirect flight muscles. **(A–F)** Projections of confocal micrographs acquired from dissected wing imaginal discs of late *SideGFP*^*Mi*00149^ third instar larvae. **(A,B)** Tile scan of a wing disc showing Side-GFP expression only in the notum (arrows). Asterisks mark the wing pouch. **(C–F)** Side-GFP largely overlaps with Twist-expressing myoblasts in wing discs. **(G)** Top: Scheme of indirect (dark grey) and direct (grey) flight muscles and the mesothoracic jump muscle (light grey, exterior view). Bottom: Tentative scheme of the innervation pattern of indirect flight muscles by the posterior dorsal mesothoracic nerve (PDMN, magenta) as seen in dissected hemi-thoraces (interior view). **(H–P)** Confocal projections of dissected hemi-thoraces stained with anti-Ankyrin2-XL antibodies of control and Side loss- and gain-of-function flies. **(H–J)** The PDMN defasciculates into five major branches that cover the entire surfaces of all six dorsal longitudinal flight muscles. Several nerve branches emanating from these fascicles sprout fine processes even between and along individual myofibrils. **(K–M)** Indirect flight muscles of *side* mutants show more secondary nerve branches compared to control flies. **(N–P)** Muscle-specific overexpression of Side results in irregular innervation of indirect flight muscles, with areas containing increased number of branches **(O)**, while others are largely devoid of nerve branches **(P)**. **(Q)** Statistical analysis of the number of terminal branches per muscle surface area (10,000 μm^2^). White frames mark regions enlarged in new micrographs in **F,I,J,L,M,O,P**. Data are means ± SD, *n* = number of hemi-thoraces. One-way ANOVA, ***P < 0.001, ns, not significant. Genotypes: *yw;*+*;*SideGFPMi00149; w*;*+*;ShGFP, w;*+*;side*^*C*137^*, ShGFP/side*^*I*1563^*, ShGFP, w;*+*;Mef2-Gal4, ShGFP/UAS-Side*^29*A*^.

In the first step, we analysed Side expression in wing imaginal disc using *SideGFP*^*Mi*00149^. We detected Side-GFPs electively in the notum of wing discs but not in the pouch (Figures 4A,B). The wing pouch develops into the dorsal and ventral wing surfaces, which do not contain any muscles or motor nerves (Bryant, 1975). In contrast, cells in the notum develop into parts of the dorsal thorax, including a subset of flight muscles and express the mesodermal marker Twist (Bate et al., 1991). Indeed, the expression domains of Twist and Side largely overlapped (Figures 4C–E). At higher magnification, *SideGFP*^*Mi*00149^ and Twist were co-expressed but did not completely overlap, probably because Twist, but not Side accumulated in the nucleus (Figure 4F).

We next examined the innervation pattern of the dorsal longitudinal muscles (DLMs), a group of six indirect flight muscles (Figure 4G) (Fernandes et al., 1991). These muscles are innervated by five exceptionally large motor neurons that together form the posterior dorsal mesothoracic nerve (PDMN), a descendent of the larval intersegmental nerve (Coggshall, 1978; Fernandes and Keshishian, 1998).

To visualise the PDMN in dissected hemithoraces, we used specific antibodies recognising *Drosophila* Ankyrin-2XL (Koch et al., 2008) (Figures 4H–P). In wild-type animals, the PDMN grew into the DLM muscle field from a ventral direction and defasciculated into evenly spaced branches projecting into anterior or posterior directions (Figures 4H–J). Compared to ShGFP controls, *side* mutant thoraces were much more fragile during preparation but showed a comparable branching pattern. However, major nerves showed increased sprouting of secondary branches compared to controls (*n* = 7) (Figures 4K–M). Evaluating their numbers on representative muscle areas (10,000 μm^2^) showed that *side* mutants had slightly more branches but this did not reach statistical significance when compared to wildtype (15 ± 3 compared to 11 ± 3 branches in controls, Figure 4Q).

Interestingly, this phenotype was strongly increased in flies overexpressing Side in muscles (*n* = 11). Muscle areas with innervation were densely covered with axonal sprouts (Figures 4N,O), whereas other areas were largely devoid of presynaptic endings (Figure 4P). Quantification confirmed that the average number of terminal branches within a randomly selected surface area (10,000 μm^2^) was highly variable in overexpressing Side flies (Figure 4Q). In areas with hyperinnervation, branch numbers were almost three-fold higher compared to controls (31 ± 16 vs. 11 ± 3 branches) (Figure 4Q). Innervation sites were generally shifted towards ventral muscle regions, leaving dorsal areas less innervated (Figure 4N). With 10 ± 2 ventral branches compared to only 1 ± 1 dorsal branch per 2.500 μm^2^ surface area, overexpressing flies showed a dramatic preference for ventral innervation sites, where PDMN axons arriving from the thoracic ganglion first hit the muscle fibres.

### Side Loss- and Gain-of-Function Causes Flight Defects

**Figure 5:**
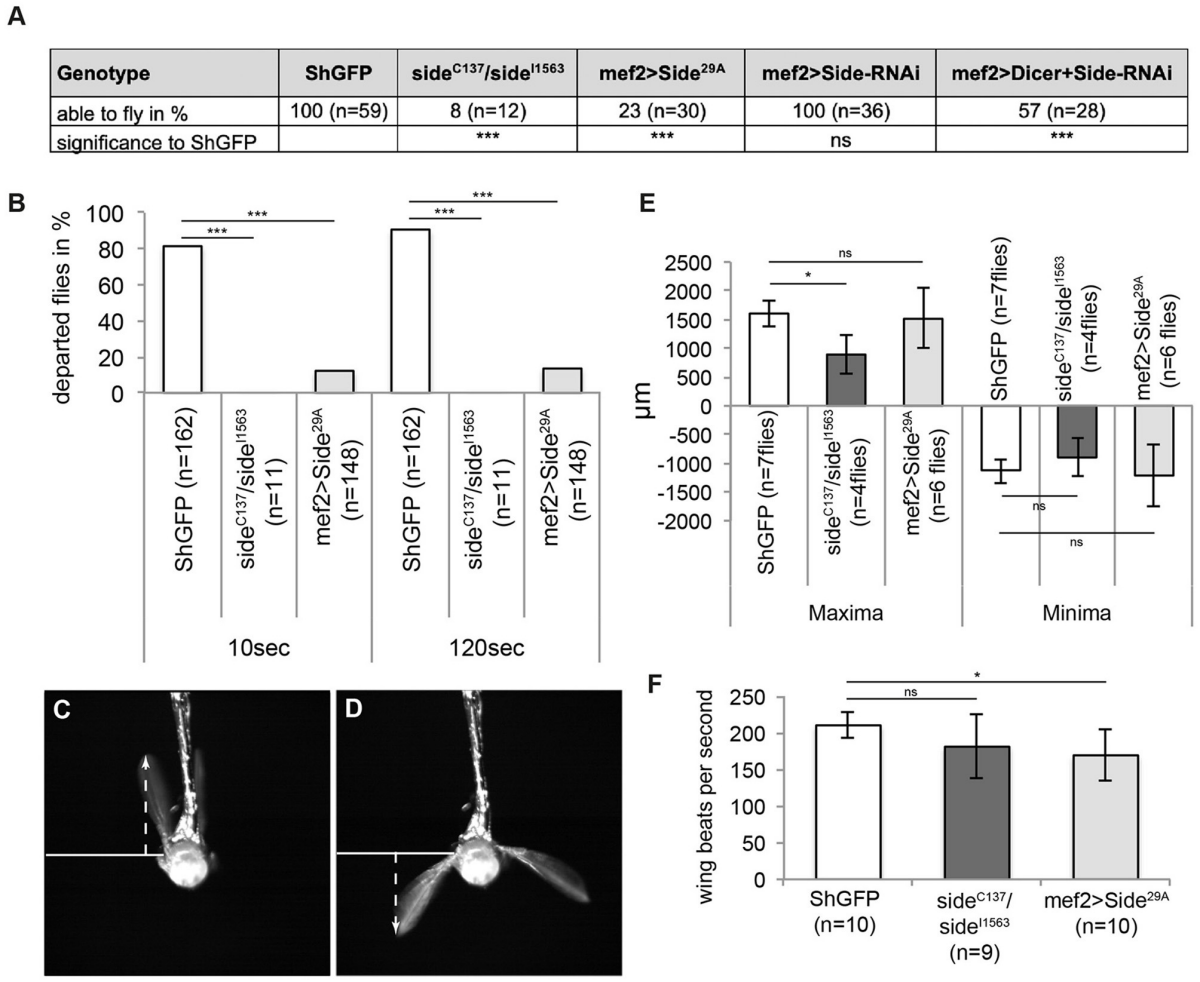
Misinnervation of indirect flight muscles results in the inability to initiate free, unrestricted flight. **(A)** *Side* mutant and overexpressing Sides are not able to fly when tested in the dropping assay. Downregulation of Side by RNAi show also reduced fly capacities compared to controls but only when enhanced by Dicer. **(B)** Percentage of flies of the indicated genotypes that depart from a platform surrounded by water (island assay). Flies lacking *side* are unable to initiate free flight, even after 120 s, whereas more than 20 and 90% of overexpressing Side and control flies, respectively, depart in the same time period. **(C,D)** Example images of high-speed video recordings during tethered flight of a *ShGFP* control fly. Arrows indicate the maximum and minimum distances from the wing hinge to the uppermost and lowermost turning points, respectively. **(E,F)** Statistical analysis of wing amplitude and wing beat. Wing amplitude is reduced in *side* mutants **(E)**, whereas overexpressing Side flies exhibit a reduced number of wing beats per second **(F)**. Data are total numbers in (**A,B**, *n* = number of males, ***P < 0.001 Fisher exact test) and means ± SD. (**E,F**, *n* = number of males, *P < 0.05, ns, not significant, one-way ANOVA). Genotypes: *w;*+*;ShGFP, w;*+*;side*^*C*137^*, ShGFP/side*^*I*1563^*, ShGFP, w;*+*;Mef2-Gal4, ShGFP/UAS-Side*^29*A*^.

Since indirect flight muscles drive the wing beat via thoracic deformations, it is difficult to judge, how innervation defects impinge on flight. It seems possible that partially innervated muscles develop sufficient force to sustain flight. However, irregular innervations might also correlate with deranged flight patterns. In initial flight tests, we dropped control and *side* mutant flies out of dry plastic vials. While all control flies immediately started flying as soon as they were airborne, most *side* mutant (92%, *n* = 12) and overexpressing Side flies (77%, *n* = 30) fell onto the substrate (Figure 5A). Similar results were obtained when Side was downregulated in developing muscles using *side*-specific dsRNA and *UAS-Dicer* under control of *Mef2-Gal4* (Figure 5A). Additionally, mutant (100%) and overexpressing (86%) flies did not depart from a solid plastic platform surrounded by a water/detergent mixture during a 2 min time interval (Figure 5B).

To find possible reasons for the inability to fly, we used a high-speed video camera to capture wing kinematics (Supplementary Movie 2). At 5,000 fps, the four main phases of the wing beat, such as upstroke, dorsal reversal, downstroke, and ventral reversal were clearly distinguishable (Figures 5C,D) (Zanker, 1990). First, we analysed the mean amplitude of wing movements in tethered flies by determining the distance between the upper and lower turning points. While *ShGFP* controls had a total amplitude of 2,744 ± 448 μm, *side* mutants achieved only 1,789 ± 583 μm and overexpressing Side flies achieved 2,727 ± 1,058 μm (Figure 5E). Depending on the strain used and the surrounding air temperature, flies move their wings at a frequency between 175 and 225 wing beats per second (wbps) (Gotz, 1968; Curtsinger and Laurie-Ahlberg, 1981; Chakraborty et al., 2015). In our analysis, tethered *ShGFP* flies showed an average frequency of 210 ± 17 wbps (Figure 5F). Wing beat frequency was significantly reduced in overexpressing Side flies (169 ± 34 wbps) but not in *side* mutants (181 ± 43 wbps) (Figure 5F). While up- and downstrokes still occurred in tethered *side* mutants, they did not seem to be powerful enough to sustain free, untethered flight. This conclusion was supported by our analysis of take-off manoeuvres. Mutant flies were unable to perform jump starts and to move their wings synchronously, preventing free, unrestricted flight (Supplementary Figure 2, Supplementary Movie 3).

### *Beat* Mutant Flies Show Misinnervation of Leg Muscles and Altered Walking Behaviour

**Figure 6:**
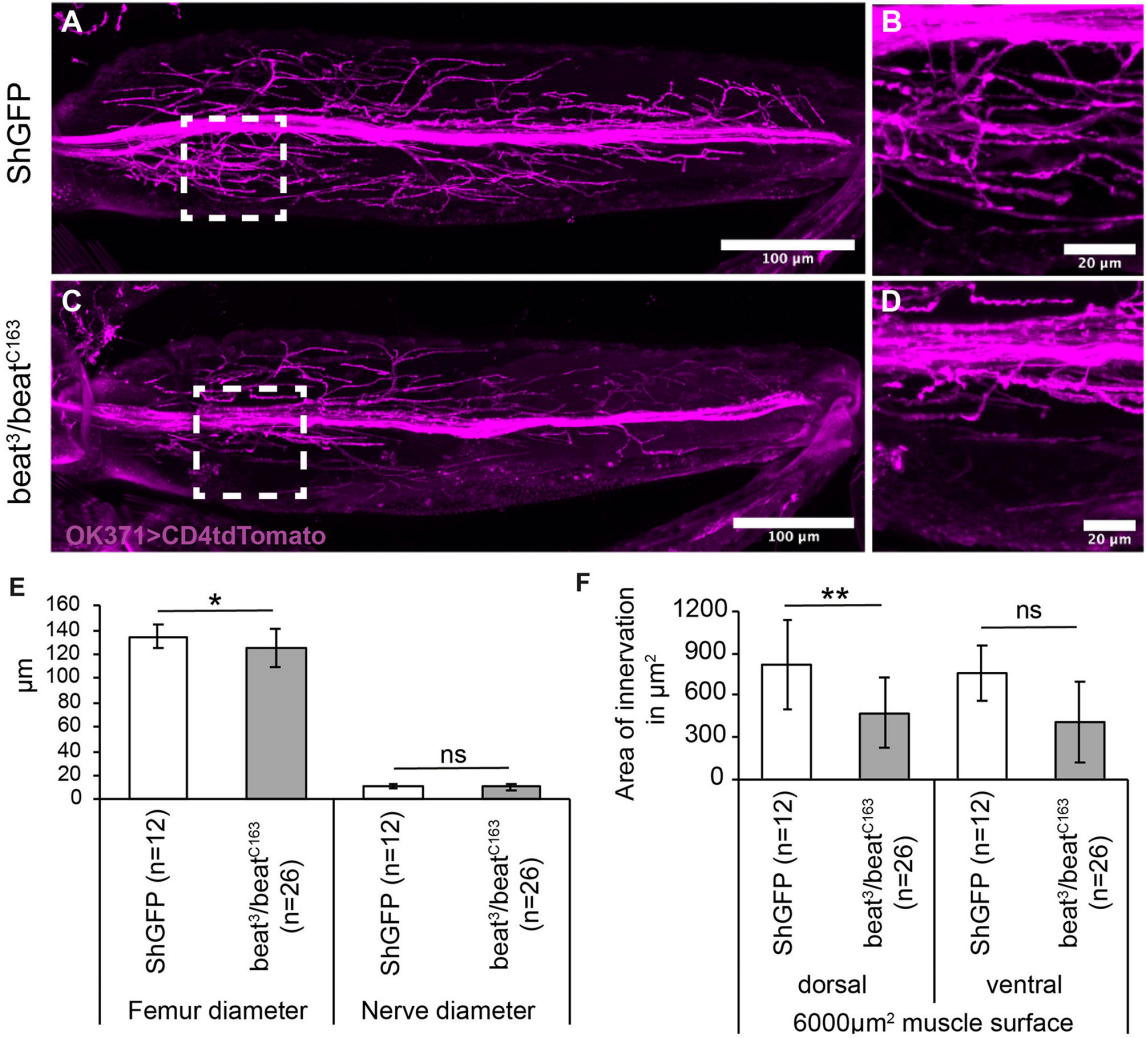
*Beat* mutant flies show innervation errors of femur muscles. **(A–D)** Confocal micrographs of motor axon projections in the femur of pharate adults expressing CD4tdTomato (magenta) under control of *OK371-Gal4*. **(A,B)** *ShGFP* control flies show axons sprouting in the periphery from a main nerve bundle. **(C,D)** In *beat* mutants, a subset of defasciculating axons does not reach peripheral muscle regions and choose innervation sites closer to the main nerve bundle. **(E,F)** Statistical analysis of femur innervation in control and *beat* mutant flies. The overall femur diameter is slightly decreased in *beat* mutants **(E)**, as is the innervation area on the dorsal tilm muscle **(F)**. White frames mark regions enlarged in new micrographs in **B,D**. Data are means ± SD and *n* = number of femurs. Two-tailed Student's *t*-test or Mann–Whitney *U*-test, **P < 0.01, *P <0.05, ns, not significant. Genotypes: *w;ShGFP;*+*, w;beat*^3^*, ShGFP/beat*^*C*163^*, ShGFP;*+*, w;OK371-Gal4, ShGFP/*+*;UAS-CD4tdTomato/*+*, w;beat*^3^*, OK371-Gal4/beat*^*C*163^*;UAS-CD4tdTomato/*+. Scale bars: **A,C** 100 μm; B, D 20 μm.

Since Beat functions as a receptor for Side, we tested also its involvement in wiring the adult motor system. To search for innervation defects, we visualised the main leg nerve using *OK371-Gal4* driving the membrane marker, CD4tdTomato. The leg nerve projected along a central trajectory through the femur in both control and in *beat* mutant flies (Figures 6A–D). Femurs and nerves were of similar thickness in controls and *beat* mutants (Figure 6E). The nerves also had a comparable number of axonal sprouts, but the defasciculated axons remained near the main nerve bundle, resulting in reduced innervation areas on the peripheral femur muscles in *beat* (473 ± 251 μm^2^) in comparison to wild-type flies (818 ± 318 μm^2^; Figure 6F).

**Figure 7:**
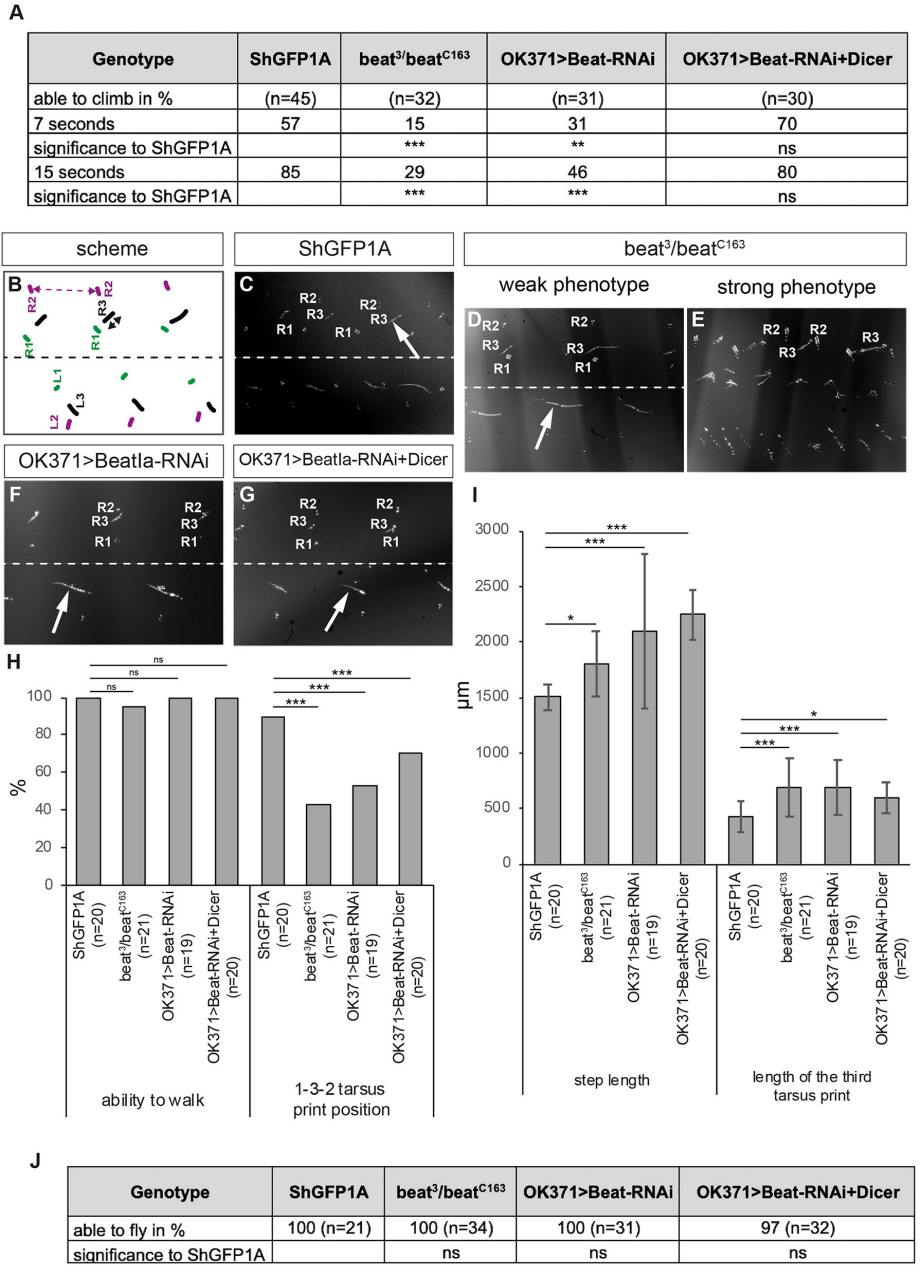
Loss of Beat alters walking behaviour. **(A)** Percentage of flies of the indicated genotypes that are able to climb in an empty vial. Loss and downregulation of Beat results in a decreased number of flies capable of climbing compared to controls. However, further enhancement using Dicer had no effect. **(B)** Scheme of tarsus print patterns. Labels as in Figure 3B. **(C–G)** Bright-field images of foot prints on glass slides coated with carbon soot. **(C)** Leg prints on the right side of *ShGFP* control flies are positioned from medial to lateral: R1, R3, R2. **(D,E)** *Beat* mutant flies show leg dragging (arrow in **D**) and crossing the midline of the first leg of the left body half **(E)**. **(F,G)** Downregulation of Beat in motoraxons by RNAi leads to weak phenotypes with a prolonged print of the third leg (arrows). **(H,I)** Statistical analysis of the leg print assay. Only 43% of *Beat* mutants show coordinated 1-3-2 leg movements **(H)**, but exhibit extended step length compared to control flies **(I)**. **(J)** All mutant and knockdown flies are able to initiate flight in the dropping assay. Data are total numbers in **H,J** and means ± SD in **A,H,I**. n= number of males. Fisher's exact test in **H,J** and one-way ANOVA in **A,H,I**, ***P < 0.001, **P < 0.01, *P< 0.05, ns, not significant. Genotypes: *w;ShGFP;*+*, w;beat*^3^*, ShGFP/beat*^*C*163^*, ShGFP;*+*, w;OK371-Gal4,ShGFP/*+*;UAS-CD4tdTomato/*+*, w;beat*^3^*,OK371-Gal4/beat*^*C*163^*;UAS-CD4tdTomato/*+, *w;OK371-Gal4/*+*;UAS-Beat-RNAi/*+*, w;OK371-Gal4/UAS-Dicer;UAS-Beat-RNAi/*+.

To test if these misinnervations also correlate with locomotion errors, we subjected *beat* knockdown and *beat* mutants to various behavioural assays. In the climbing assay, significantly less number of transheterozygous *beat* mutant and knockdown flies were able to climb the steep plastic vials compared to controls (Figure 7A). However, enhancing presynaptic knockdown using Dicer had no effect (Figure 7A).

Similar results were also obtained in the leg print assay (Figures 7B–I). While *beat*^3^/*beat*^*C*163^ adults showed strong leg coordination phenotypes scompared to ShGFP controls (Figures 7B–E), knockdown of *beat* function using OK371-Gal4 with and without Dicer had only little effects (Figures 7F,G). In addition, approximately half of *beat* mutant flies (43%) maintained the T1-T3-T2 tarsus print positions (Figure 7H) but display significant changes in T2 step length (1,802 ± 291μm vs. 1,506 ± 118 μm in *ShGFP* controls) (Figure 7I). Analysing the length of the metathoracic leg print showed an ~1.5-fold increase in both *beat* mutants and RNAi flies in comparison to control flies (685 ± 265 μm in *beat* mutants vs. 422 ± 142 μm in ShGFP) (Figure 7I). To test flight abilities, we performed our dropping assay. In contrast to *side* mutant flies, all *beat* mutants were able to fly after being dropped out of a vial (Figure 7J).

These experiments together with the innervation defects indicate that *beat* mutant flies have a weaker phenotype compared to *side* mutants, suggesting that Beat might not act as the sole receptor for Side during metamorphosis.

## Discussion

In this paper, we show that embryonic guidance cues are also employed during metamorphosis for wiring a completely different set of adult muscles, and that even loss of *sidestep* (*side*), which produces one of the strongest miswiring phenotypes in motor axons currently known, is compatible with viability. Side was originally characterised during embryonic development, when motor axons first exit the CNS to target peripheral muscles (Sink et al., 2001). Genetic and biochemical analyses suggest that Side functions as a substrate-bound protein that effectively attracts motor axons (Sink et al., 2001; de Jong et al., 2005; Siebert et al., 2009). Overexpression of Side in *side* mutant backgrounds demonstrated that growth cones are very well able to distinguish between Side-labelled and Side-unlabelled substrates (Siebert et al., 2009). The expression pattern is hence an important determinant factor for pathway choices elicited by this potent contact attractant.

The Side protein family is highly conserved in arthropods and contains eight members in *Drosophila* (Aberle, 2009; Li et al., 2017). Their expression pattern is consistent with potential functions as substrate labels (Li et al., 2017). Since Side protein is no longer detectable in late embryos using immunostainings (Sink et al., 2001; Siebert et al., 2009), we wanted to cheque if it is re-expressed during metamorphosis or if it is functionally replaced by one of its paralogs. To examine its postembryonic expression, we took advantage of a fluorescent exon-trap line that fuses GFP with the cytoplasmic domain of endogenous Side (Nagarkar-Jaiswal et al., 2015). In leg imaginal discs, Side was already expressed in Zfh1-positive adult muscle precursors in late third instar larvae, when adult motor axons begin to grow out of the CNS. In contrast to embryonic development, where Side was strongly expressed in sensory neurons (Sink et al., 2001; Siebert et al., 2009), we did not detect it in sensory neurons of imaginal discs. Since embryonic motor axons fasciculate with afferent sensory axons (Siebert et al., 2009), a similar mechanism seemed plausible for adult leg nerves as sensory neurons in the disc project towards the adult brain. However, motor axons have already reached the distal-most end of the disc before sensory neurons even sprout axons (Brierley et al., 2012; Venkatasubramanian et al., 2019). Instead, motor axons might contact Side-expressing myoblasts. The developing primary and secondary branches seem to keep steady contact with developing muscles (Venkatasubramanian et al., 2019). Thus, in legs, the development of adult motor nerves seems to differ from embryonic mechanisms as such that motor axons contact Side-expressing muscle precursors but not Side-positive sensory axons.

The specific expression of Side in leg and wing imaginal discs suggests that Side is likely to exert major guidance functions during metamorphosis and is likely not replaced by one of its paralogs. In this respect, it is interesting to note that leg muscles develop *de novo* from undifferentiated precursors. This resembles the situation in embryos, where undifferentiated myoblasts fuse with founder cells to generate multinuclear muscle fibres. Due to these developmental similarities, embryonic guidance pathways might be reactivated to establish adult wiring patterns.

In the absence of *side*, leg nerves were overtly fasciculated and projected along aberrant pathways. Consequently, leg muscles partially or completely lacked NMJs, correlating within the inability to execute coordinated walking on flat substrates.

Leg-based behaviours were also impaired in the muscles of overexpressing Side flies, but overexpression had even more drastic consequences for indirect flight muscles. Axonal growth across the array of DLMs was generally limited and branching at ventral nerve entry sites was overtly increased. Terminal nerve branches remained largely superficial and were less able to reach deep between neighbouring myofibrils in comparison to wild-type motor branches. Despite the fact that most DLMs received innervation, it was apparently insufficient to trigger unrestrained flight from a small platform in the island assay. Since DLMs control the downstroke of wing, it is possible that irregular innervation develops insufficient uplift.

DLM innervation in transheterozygous *side* mutants appeared surprisingly normal in our histological examinations with only a slight increase in terminal branching. Nevertheless, these flies were completely unable to freely depart from a horizontal platform. A possible explanation for this observation could be that the indirect dorsoventral flight muscles or the synchronous direct flight muscles are more strongly affected in *side* mutants. In fact, we found that Side is strongly expressed in progenitor cells in the notum of wing discs that develop into these muscle fibres during normal development (Muller et al., 2010).

In comparison to *side, beat* mutant flies show considerably weaker phenotypes in both the innervation pattern of femur muscles and in different locomotion assays. In the absence of *beat* function, flies are quite able to walk on flat substrates but do show evident dragging and mispositioning phenotypes. Nevertheless, *beat* mutants show a higher viability and agility compared to *side* mutants, suggesting that Beat is not involved in all Side functions during metamorphosis and might function in concert with other members of the Beat family.

Our results also allow for more general conclusions. First, miswiring of motor nerves disturbs locomotor behaviours but does not abolish them completely in the paradigms examined (walking, flying and grooming). The neuromuscular system and/or central pattern generators seem to possess the ability to compensate for the loss of NMJs to a certain extent. Movements probably fail only when a certain threshold of non-innervated muscles is surpassed. Second, defects in locomotion can be caused by irregular wiring of the somatic musculature and do not need to arise in higher CNS centres, as overexpression of Side, specifically in muscles, was sufficient to trigger dysfunction. Third, misguided axons can cause unilateral or asymmetric movement impairements, occurring only on one side of the body. And last, even strong and permanent neuromuscular innervation defects are compatible with viability, permitting examination and genetic dissection of postembryonic circuits and behaviours.

## Data Availability Statement

The original contributions presented in the study are included in the article/Supplementary Material, further inquiries can be directed to the corresponding author/s.

## Author Contributions

JK and MB: methodology, data curation, and imaging. JK and HA: writing. HA: supervision, project administration, and funding acquisition. All authors contributed to the article and approved the submitted version.

## Conflict of Interest

The authors declare that the research was conducted in the absence of any commercial or financial relationships that could be construed as a potential conflict of interest.

## Supplementary Material

Supplementary Figure 1 Side mutant flies are unable to execute coordinated grooming behaviours. **(A,B)** Tile scan of CD4tdTomato-labelled leg nerves (magenta) in homozygous *side* mutant pharate adults. The main leg nerve is dislocated towards an extreme ventral position. White frames mark regions enlarged in **B**. **(C–N)** Representative images of flies of the indicated genotypes coated with Reactive Yellow 86 dust at 0, 4, 30, and 60 min after complete dusting. **(C–F)** *ShGFP* flies clean their eyes within 4 min (arrow in **D**), their head capsule within 30 min (arrow in **E**), and their abdomen and wings within 60 min (arrowhead in **F**). **(G–J)** Forty percent of *side* mutant flies are able to clean their eyes during the first 4 min of the assay (arrow in **H**). However, head capsule (arrow in **I**), wings (arrowhead in **J**), and abdomen remain covered with dust after 60 min. **(K–N)** Flies overexpressing Side in muscles require 30 min to clean their eyes (arrow in **M**) and 60 min to clean their head capsule (arrow in **N**), but fail to clean their wings (arrowhead in **N**). **(O)** Statistical analysis of grooming behaviour of indicated genotypes. Genotypes: *w;OK371-Gal4/*+*;side*^*C*137^*, ShGFP/side*^*I*1563^*, ShGFP, UAS-CD4tdTomato, w;*+*;ShGFP, w;*+*;side*^*C*137^*, ShGFP/side*^*I*1563^*, ShGFP, w;*+*;Mef2-Gal4, ShGFP/UAS-Side*^29*A*^. Scale bars: A 50 μm, B 20 μm.

Supplementary Figure 2 S*ide* mutant and overexpressing flies show an altered take-off sequence. **(A–R)** Time series showing flies of the indicated genotypes during flight initiation using high-speed videography. **(A–F)** *ShGFP* fly taking-off from a foamed rubber substrate. Flight starts with raising the wings high above the body **(B,C)**. The fly then lifts the first and third legs before the wings strike downward **(D)**. Take-off is triggered by jumping with the second pair of legs **(E,F)**. **(G–L)** Fly lacking *side* initiates flight from a plastic substrate by hardly lifting its wings **(G)** and immediately pushing them downward even before any leg is released from the ground **(H)**. The body is raised above the substrate only during the second wing beat **(I)**. Failures to synchronise wing stroke with jumping behaviour results in rotation around the body axis **(J–L)**. **(M–R)** Fly overexpressing Side in muscles initiates flight from a plastic substrate by raising the wings insufficiently **(M,N)**. During the following downstroke **(O)**, legs jump off asymmetrically, with legs on the left side being delayed **(P–Q)**, resulting in a rotation around the anterior-posterior axis **(R)**. Arrows, left wing in all images; arrowheads, first left leg; asterisks, start position of second left leg. Genotypes: *w;*+*;ShGFP, w;*+*;side*^*C*137^*, ShGFP/side*^*I*1563^*, ShGFP, w;*+*;Mef2-Gal4, ShGFP/UAS-Side*^29*A*^.

Supplementary Figure 3 Characterisation of the *side* exon trap line and *side* downregulation by RNAi. **(A)** The exon trap line Mi00149-GFSTF (SideGFP^Mi00149^) is inserted between exon 16 and 17 of the *side* locus, resulting in the insertion of GFP at amino acid 918 of endogenous Side. Ex, exon; Sp, signal petide; Ig, immunoglobulin domain; FNIII fibronectin type-III domain; TM, transmembrane domain. Not to scale. **(B,C)** ShGFP control and SideGFP^Mi00149^ embryos (st.16) immunostainings using anti-FasII antibodies. ISNb nerves (arrows) branch regularly into ventral muscles fields. **(D,E)** Stitched confocal micrographs of dissected third instar larvae stained with anti-Discs large (Dlg, green) antibodies and phalloidin coupled to Texas Red (red). Neuromuscular junctions (NMJs) are present at expected positions in both control and SideGFP^Mi00149^ larvae. Arrows mark NMJs on muscle 12 in three consecutive segments. **(F–G)** Quantitative evaluation of the ability to fly in the dropping assay. **(H–J)** Confocal images of embryos (st.14) stained with anti-Side antibodies. Expression of a transgenic *side* RNAi construct in all postmitotic neurons using elav-Gal4 diminishes Side proteins starting at stage 14. Downregulation is increased by co-expression of Dicer. Genotypes: *w;*+*;ShGFP, yw;*+*;SideGFP*^*Mi*00149^, *w;UAS-Side-RNAi/*+*;elav-Gal4/*+*, w;UAS-Side-RNAi/*+*;elav-Gal4/UAS-Dicer*. Scale bars: B–C25 μm, D–E 200 μm, H–J 20 μm.

Supplementary Movie 1 Miswiring alters locomotion patterns. Flies walking freely over glass slides recorded from ventral views using a high-speed video camera (frame rate of 125 fps). While *ShGFP* control flies use an alternating tripod gait, *side* mutants display an aberrant gait, including difficulties in leg coordination, spatial positioning, and chronology. Flies overexpressing Side in muscles show predominantly tripod-like patterns but occasionally limp with their metathoracic legs. Genotypes in all movies: *w;*+*;ShGFP, w;*+*;side*^*C*137^*, ShGFP/side*^*I*1563^*, ShGFP, w;*+*;Mef2-Gal4, ShGFP/UAS-Side*^29*A*^.

Supplementary Movie 2 Side-induced miswiring disrupts wing-haltere coordination. While wings and halteres move in strict antiphase and maximal amplitude in *ShGFP* control flies, a subset of *side* mutant flies show reduced wing amplitudes and only minimal haltere movements. Halteres in flies overexpressing Side in muscles occasionally do not move at all despite pronounced wing amplitudes. Flight was recorded in tethered flies using a high-speed video camera set at 5,000 fps.

Supplementary Movie 3 Uncontrolled take-off behaviours in *side* mutant flies. *ShGFP* control flies jump with their mesothoracic legs to lift off the substrate and initiate free flight, when the initial wing stroke reaches a ventral turning point. Take-off is severely compromised in flies lacking *side* or overexpressing it in muscles, as no or non-synchronic jumps tend to imbalance the flies. Note, aberrant leg postures but quite normal haltere movements during take-off. Foamed rubber or plastic substrates were used during the recordings (5,000 fps).
