# Supplementary figures and images for: Misregulation of Drosophila Sidestep Leads to Uncontrolled Wiring of the Adult Neuromuscular System and Severe Locomotion Defects
Source: Front Neural Circuits. 2021 Jun 3;15:658791. doi: 10.3389/fncir.2021.658791 (PMC8209334; doi:10.3389/fncir.2021.658791)

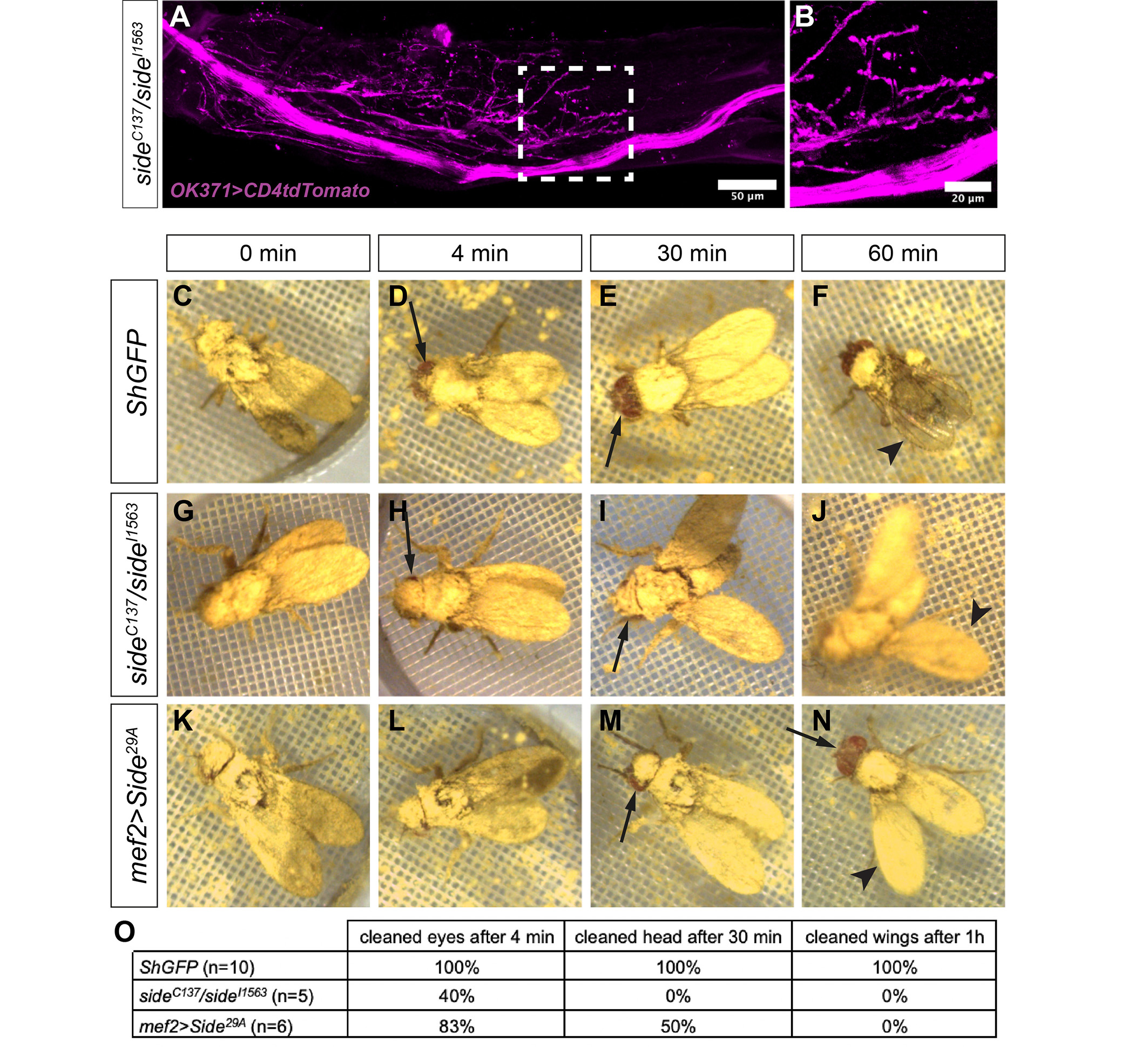

Supplement: Supplementary Figure 1 — Side mutant flies are unable to execute coordinated grooming behaviours. (A,B) Tile scan of CD4tdTomato-labelled leg nerves (magenta) in homozygous side mutant pharate adults. The main leg nerve is dislocated towards an extreme ventral position. White frames mark regions enlarged in B. (C–N) Representative images of flies of the indicated genotypes coated with Reactive Yellow 86 dust at 0, 4, 30, and 60 min after complete dusting. (C–F) ShGFP flies clean their eyes within 4 min (arrow in D), their head capsule within 30 min (arrow in E), and their abdomen and wings within 60 min (arrowhead in F). (G–J) Forty percent of side mutant flies are able to clean their eyes during the first 4 min of the assay (arrow in H). However, head capsule (arrow in I), wings (arrowhead in J), and abdomen remain covered with dust after 60 min. (K–N) Flies overexpressing Side in muscles require 30 min to clean their eyes (arrow in M) and 60 min to clean their head capsule (arrow in N), but fail to clean their wings (arrowhead in N). (O) Statistical analysis of grooming behaviour of indicated genotypes. Genotypes: w;OK371-Gal4/+;sideC137, ShGFP/sideI1563, ShGFP, UAS-CD4tdTomato, w;+;ShGFP, w;+;sideC137, ShGFP/sideI1563, ShGFP, w;+;Mef2-Gal4, ShGFP/UAS-Side29A. Scale bars: A 50 μm, B 20 μm. [file Image_1.JPEG]

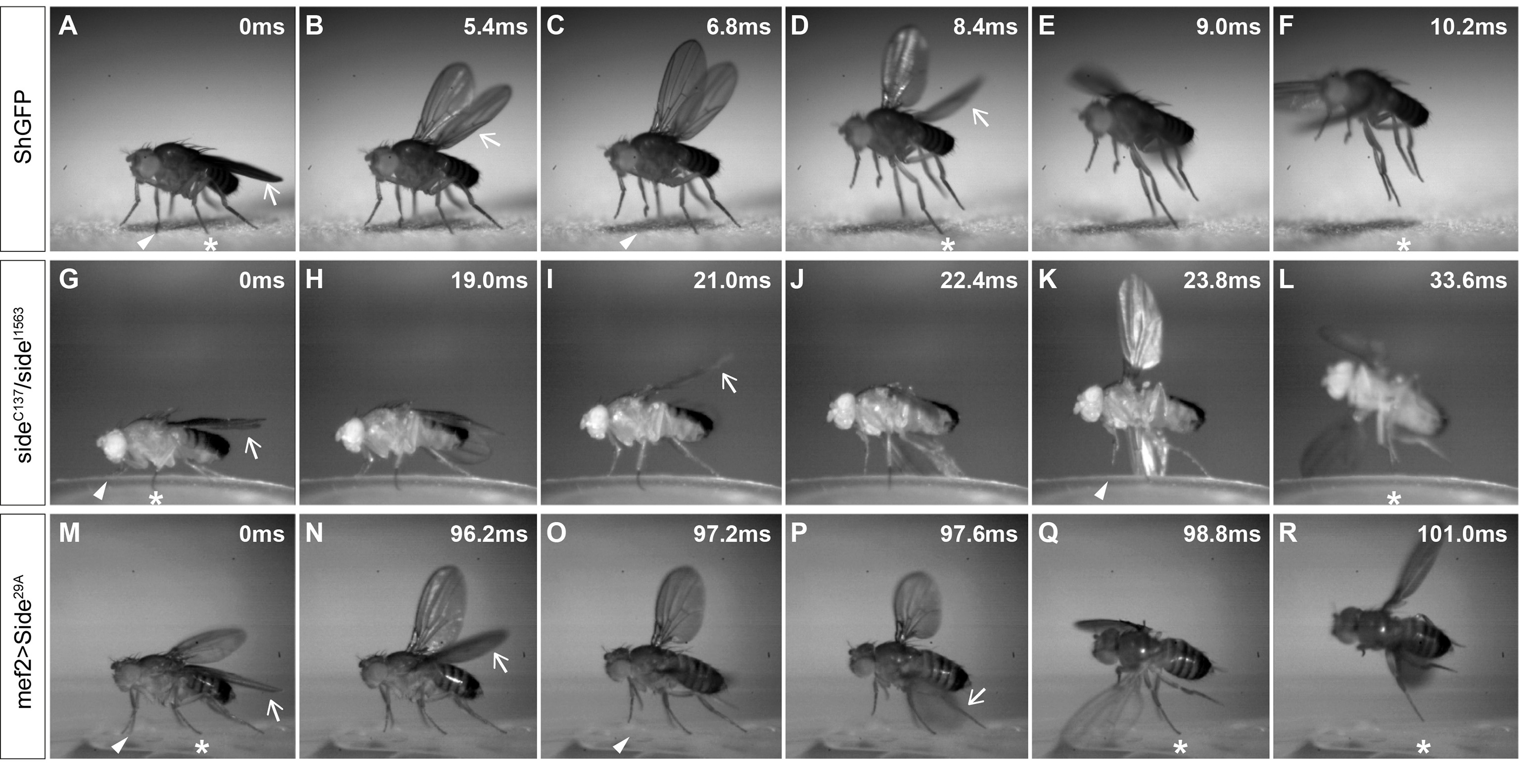

Supplement: Supplementary Figure 2 — Side mutant and overexpressing flies show an altered take-off sequence. (A–R) Time series showing flies of the indicated genotypes during flight initiation using high-speed videography. (A–F) ShGFP fly taking-off from a foamed rubber substrate. Flight starts with raising the wings high above the body (B,C). The fly then lifts the first and third legs before the wings strike downward (D). Take-off is triggered by jumping with the second pair of legs (E,F). (G–L) Fly lacking side initiates flight from a plastic substrate by hardly lifting its wings (G) and immediately pushing them downward even before any leg is released from the ground (H). The body is raised above the substrate only during the second wing beat (I). Failures to synchronise wing stroke with jumping behaviour results in rotation around the body axis (J–L). (M–R) Fly overexpressing Side in muscles initiates flight from a plastic substrate by raising the wings insufficiently (M,N). During the following downstroke (O), legs jump off asymmetrically, with legs on the left side being delayed (P–Q), resulting in a rotation around the anterior-posterior axis (R). Arrows, left wing in all images; arrowheads, first left leg; asterisks, start position of second left leg. Genotypes: w;+;ShGFP, w;+;sideC137, ShGFP/sideI1563, ShGFP, w;+;Mef2-Gal4, ShGFP/UAS-Side29A. [file Image_2.JPEG]

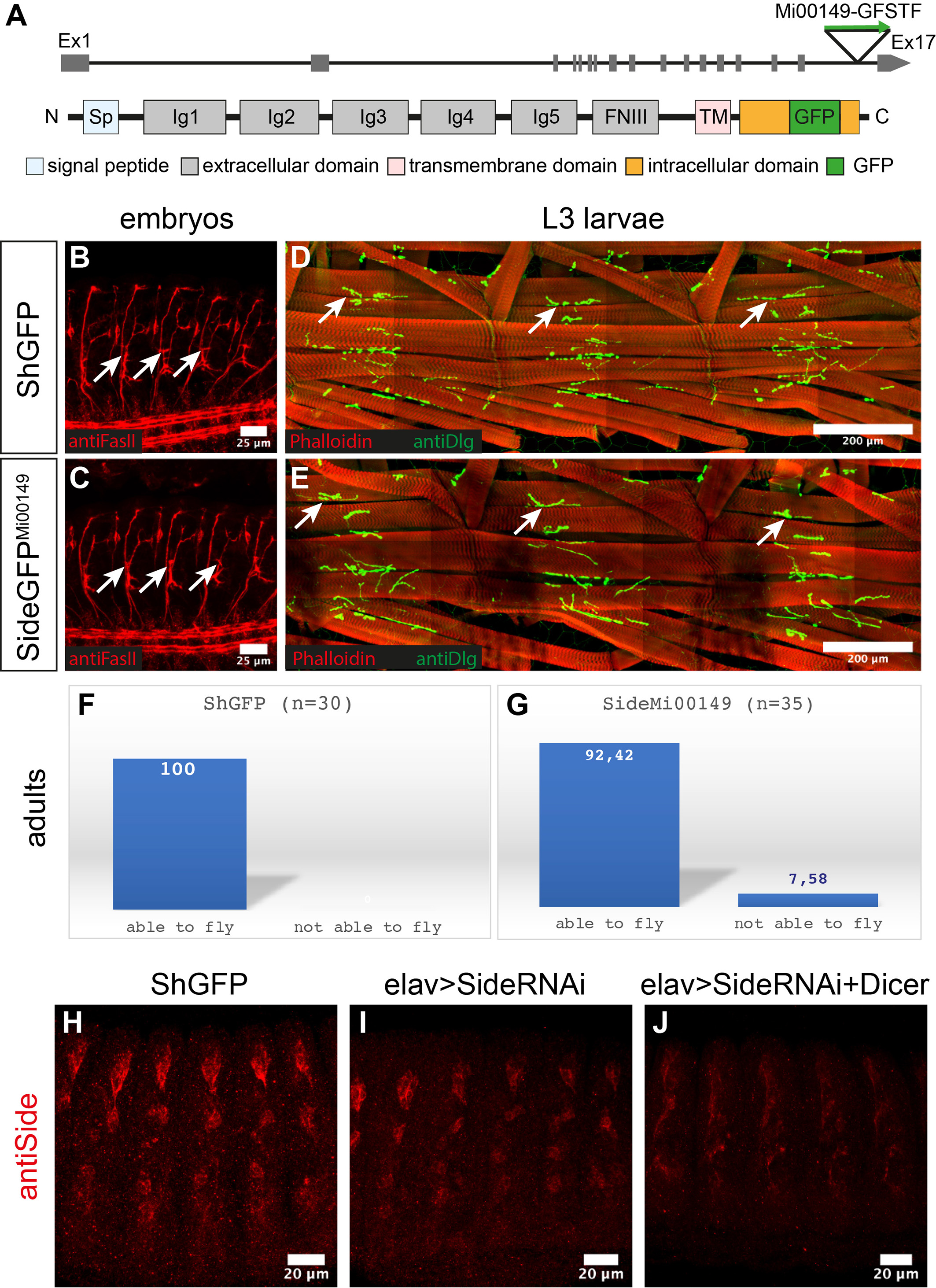

Supplement: Supplementary Figure 3 — Characterisation of the side exon trap line and side downregulation by RNAi. (A) The exon trap line Mi00149-GFSTF (SideGFPMi00149) is inserted between exon 16 and 17 of the side locus, resulting in the insertion of GFP at amino acid 918 of endogenous Side. Ex, exon; Sp, signal petide; Ig, immunoglobulin domain; FNIII fibronectin type-III domain; TM, transmembrane domain. Not to scale. (B,C) ShGFP control and SideGFPMi00149 embryos (st.16) immunostainings using anti-FasII antibodies. ISNb nerves (arrows) branch regularly into ventral muscles fields. (D,E) Stitched confocal micrographs of dissected third instar larvae stained with anti-Discs large (Dlg, green) antibodies and phalloidin coupled to Texas Red (red). Neuromuscular junctions (NMJs) are present at expected positions in both control and SideGFPMi00149 larvae. Arrows mark NMJs on muscle 12 in three consecutive segments. (F–G) Quantitative evaluation of the ability to fly in the dropping assay. (H–J) Confocal images of embryos (st.14) stained with anti-Side antibodies. Expression of a transgenic side RNAi construct in all postmitotic neurons using elav-Gal4 diminishes Side proteins starting at stage 14. Downregulation is increased by co-expression of Dicer. Genotypes: w;+;ShGFP, yw;+;SideGFPMi00149, w;UAS-Side-RNAi/+;elav-Gal4/+, w;UAS-Side-RNAi/+;elav-Gal4/UAS-Dicer. Scale bars: B–C25 μm, D–E 200 μm, H–J 20 μm. [file Image_3.JPEG]
